# Well-Defined Glycopolymer Chitosan Mimics for Design of Chitosan Nanocomposites

**DOI:** 10.1021/acs.biomac.5c01270

**Published:** 2025-10-23

**Authors:** Toby R. Edwards, Penelope E. Jankoski, Latoyia P. Downs, Musa Rabiu, Lisa K. Kemp, Travis L. Thornell, Dane N. Wedgeworth, J. Kent Newman, Tristan D. Clemons, Shahid Karim, Sarah E. Morgan

**Affiliations:** 1 School of Polymer Science and Engineering, 5104 University of Southern Mississippi, 118 College Drive, #5050, Hattiesburg, Mississippi 39406, United States; ‡ School of Biological, Environmental, and Earth Sciences, 5104 University of Southern Mississippi, 118 College Drive, #5018, Hattiesburg, Mississippi 39180, United States; 3 U.S. Army Engineer Research and Development Center, 3909 Halls Ferry Road, Vicksburg, Mississippi 39180, United States

## Abstract

Chitosan, a naturally derived polysaccharide with intriguing antimicrobial and polycationic properties, is highly desirable as a biosourced and biodegradable material for biomedical, food packaging, and personal care applications. Its inherent high levels of variability in molecular weight, dispersity, and degree of deacetylation, however, make the establishment of structure–property–processing relationships difficult and limit materials development. In this study, a novel methacrylate-based glycomonomer with saccharide structure similar to that of chitosan was synthesized and copolymerized with methyl methacrylate via reversible addition–fragmentation chain-transfer (RAFT) polymerization to create a series of well-defined chitosan mimics with controlled molecular weights and low dispersity (<1.1). Evaluation of mammalian cytotoxicity and antibacterial activity against *Escherichia coli* and *Staphylococcus aureus* revealed performance similar to that of chitosan. The copolymers were used as models to evaluate difficult-to-probe interactions between chitosan and graphene oxide (GO) and elucidate mechanisms of mechanical property improvements observed in chitosan/GO nanocomposite films.

## Graphical abstract

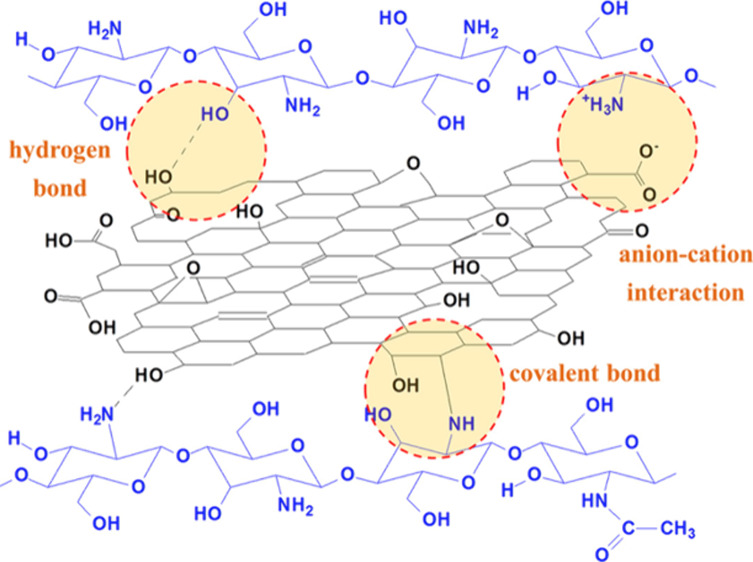

## Introduction

Chitosan is a biopolymer formed from the deacetylation (greater than 50%) of naturally abundant chitin (poly­(*N*-acetylglucosamine)) and is the copolymer of glucosamine (2-amino-2-deoxy-β-d-glucopyranose) and N-acetylglucosamine (2-acetamide-2-deoxy-β-d-glucopyranose).^1^ It can be easily protonated in acidic environments to provide antibacterial and antifungal properties while maintaining low cytotoxicity.^2,3^ These properties have been studied and leveraged for applications in the medical, agricultural, water purification, and packaging industries.^4–6^ The molecular weight, dispersity, and degree of deacetylation (DD) vary widely in commercially available chitosan grades, and these features affect solubility, tensile strength, toughness, and crystallization.^7,8^ The variability of chitosan is one of the major challenges in defining structure/property relationships to enable materials development for specific applications. To address this challenge, research efforts have been devoted to developing well characterized synthetic chitosan mimics using ring-opening,^9^ enzymatic,^10^ cationic,^11,12^ and radical polymerization.^13^ Liau et al. demonstrated the synthesis of a glycopolymer chitosan mimic with similar antibacterial properties to chitosan using free radical polymerization coupled with fractional precipitation to achieve desired molecular weights with low dispersity.^13^ However, they reported that attempts to polymerize their monomer, 1-methyl N-Fmoc-6-acryloyl-β-d-glucosaminoside, via reversible addition–fragmentation chain transfer (RAFT) techniques were unsuccessful. Further control using RAFT polymerization is desirable, as it allows polymerization of water-soluble bioinspired polymers with controlled molecular weights and low dispersity.

The mechanical, electrical, and thermal properties of chitosan can be improved through the addition of graphene oxide (GO) additives, which are commonly used because the oxygen functionality aids in dispersion and promotes interactions with polar functional groups.^14^ Commercial GO also has several sources of variability, such as the size of the particles and the amount of oxidation on the surface. These examples of variation highlight some of the challenges associated with studying such materials, where the terms “chitosan” and “graphene oxide” are used to describe materials with an array of physical and chemical attributes.

Recent research reports of chitosan/GO nanocomposites have targeted applications including wastewater treatment,^15,16^ filtration membranes,^17,18^ biosensing,^19–21^ tissue engineering,^22,23^ drug delivery,^24^ and food packaging.^25^ However, due to the variability associated with both GO and chitosan and the large number of possible formulations of chitosan/GO composites, measured properties vary widely and reported trends often appear inconsistent. For example, some researchers have reported that the addition of GO to chitosan improves both toughness and strength;^24,26–28^ while others observed an increase in tensile strength and Young’s modulus but an overall reduction in toughness.^25,29^ Several GO variables have been evaluated independently for their effects on chitosan properties. For example, Abolhassani et al. evaluated chitosan composite films with nano- and microscale GO and reported that crystallinity, bonding behavior, surface/fracture morphology, and tensile behavior depended on the size of the GO particle, with nano-GO yielding lower crystallinity, reduced binding to organic dye, a finer morphology, and increased tensile elongation in comparison to the micro-GO blend.^16^ Han Lyn et al. evaluated the impact of GO reduction in chitosan blends, and reported an increase in the strength of the films with an increase in GO carbon/oxygen ratio.^30^ There remains a lack of understanding of the complex interactions between inherently variable chitosan and GO and their influence on nanocomposite properties.

In this work, well-defined synthetic mimics of chitosan were prepared and blended with graphene oxide of different sizes and oxidation levels to systematically evaluate the effects of the additives and their interactions on the properties of the nanocomposites. A RAFT-polymerizable saccharide-containing monomer, methyl *N*-Boc-6-methacryloyl-β-d-glucosaminoside (MBMG) was synthesized and characterized. Novel cationic methacrylate-based glyco-copolymers with pendant saccharides were designed to mimic chitosan at two molecular weights and degrees of deacetylation, allowing evaluation of the independent variables and their interactions. The primary amines of the synthetic mimics are similar chemically and sterically to the d-glucosamine repeat units of chitosan, providing models to evaluate amine interactions with GO via X-ray photoelectron spectroscopy (XPS). Cytotoxicity and antibacterial properties of the four new mimics were evaluated and compared to those of chitosan. Chitosan/GO films were prepared, and their physical properties were evaluated.

## Methods and Materials

### Materials

Glucosamine hydrochloride (98%), acetyl bromide (99%), anhydrous methanol (99.8%), pyridine, acetyl chloride (AcCl) (98%), methanol-*d*_4_ (MeOH-*d*_4_, 99 atom % D), deuterium oxide (99.9 atom % D), dimethyl sulfoxide-*d*_6_ (99.9 atom % D), sodium bicarbonate, di-*tert*-butyl dicarbonate (boc2O, 99%), lipase acrylic resin from candida antarctica, vinyl methacrylate (98%), anhydrous dimethyl sulfoxide (DMSO, 99.9%), chitosan (CH) with 75–85% deacetylation of medium molecular weight (190–310 kDa) and low molecular weight (50–190 kDa) 75–85%, Dulbecco’s Modified Eagle Medium (DMEM), and 2,2’-azobis­(2-methylpropionitrile) (AIBN) were purchased from Sigma-Aldrich. AIBN was purified via recrystallization in methanol. 4-cyano [(dodecylsulfanylthiocarbonyl) sulfanyl] pentanoic acid (CDP) was purchased from Boron Molecular and used without further purification. Methyl methacrylate was purchased from Sigma-Aldrich, and the stabilizer was removed via a prepacked hydroquinone inhibitor removal column also purchased from Sigma-Aldrich. Triton X-100 and phosphate-buffered saline (PBS) were purchased from Sigma-Aldrich, LH Lithium Heparin tubes (3 mL) were purchased from Greiner Bio-One, 96-well polypropylene PCR plate purchased from VWR, and 96-well plate with flat, transparent bottom purchased from Anicrin. *Escherichia coli (E. coli*; strain: DH5a) and *Staphylococcus aureus* (*S. aureus*) (strain: RN4220) were utilized from frozen stock, and Luria–Bertani (LB) agar plates were purchased from Sigma-Aldrich. GO samples of different diameters were purchased as dispersions in water from Graphene Supermarket (90–120 nm diameter sample concentration was 1 mg/mL and the 0.5–5 μm diameter sample concentration was 5 mg/mL). All solvents were reagent grade, purchased from Sigma-Aldrich, and used without further purification. Acetate buffer (pH 4, 0.1M) was prepared with acetic acid and sodium acetate.

### Synthesis of Methyl *N*-Boc-6-methacryloyl-β-d-glucosaminoside (MBMG)

Methyl β-d-glucosaminoside was synthesized as previously reported and characterized by ^1^H NMR (Figure S1).^31^ Methyl β-d-glucosaminoside (1 g, 4.3 mmol) was dissolved in a solution of methanol (10 mL), triethylamine (7 mL), and di-*tert*-butyl dicarbonate anhydride (2.1 g, 9.5 mmol) and stirred for 48 h at 55 °C. The methanol was removed via a rotary evaporator, and the solid methyl *N*-Boc-β-d-glucosaminoside product was dried overnight in a vacuum oven at 50 °C and characterized by ^1^H NMR (Figure S2). Methyl *N*-Boc-β-d-glucosaminoside (1 g, 3.6 mmol) was dissolved in *tert*-butyl alcohol (60 mL) at 60 °C using a heating block and a shaker plate. Vinyl methacrylate (0.842 mL, 7.2 mmol), immobilized lipase (1 g), and pyridine (5 mL) were added, and the flask was shaken at 1800 rpm for 72 h at 55 °C. The reaction mixture was dried to a dark brown paste using rotary evaporation. The paste was dissolved in 90:10 ethyl acetate:hexane and purified via flash chromatography with a stationary phase of 60 Å packed silica. The column fractions containing the MBMG were dried in vacuum oven at 40 °C to a light brown solid and characterized via ^13^C NMR and ^1^H NMR spectroscopy (Figure S3a,b).

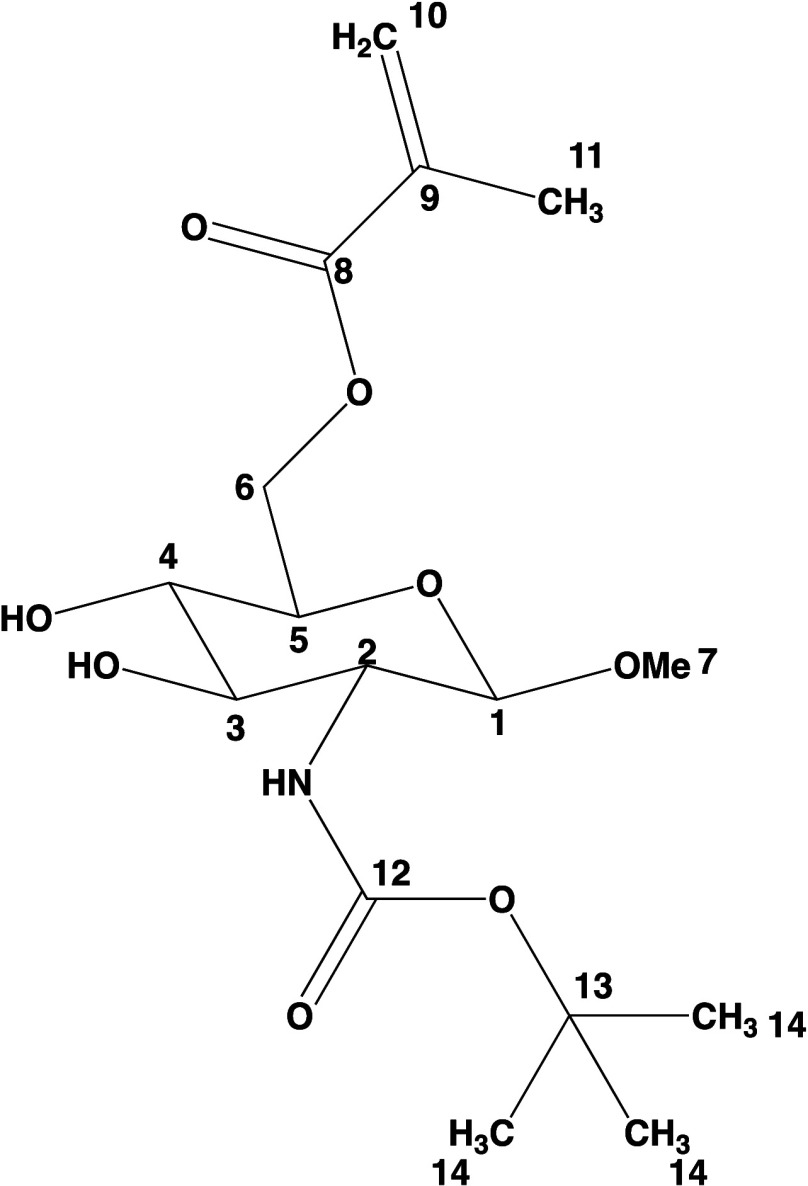



^**1**^ **H NMR** (600 MHz, MeOH-d4) δ6.15 (s, ^1^H, CH2 = C), 5.66 (s, ^1^H, CH2 = C), 4.51 (d, 1H, C1), 4.30 (m, 2H, C6), 3.51 (s, 3H, CH3-O), 3.32–3.52 (m, 4H, (C2,C3,C4,C5)), 1.97 (s, 3H, CH3-C), 1.47 (s, 9H, t-boc) ^**13**^ **C NMR** (150 MHz, MeOH-d4): 167.29­(C8), 157.2­(C12), 136.32 (C9) 136.14 (10), 124.89 (C13), 102.80­(C1), 74.67­(C3), 73.88­(C4), 70.91­(C5), 63.59­(C6), 55.60­(C2), 48.10­(C7), 27.35­(C14), 16.97­(C11)

### Polymerization of MBMG

#### Homopolymerization

Low (93 kDa) and high (162 kDa) molecular weight homopolymer chitosan mimics were prepared using RAFT polymerization with AIBN as the thermal initiator and CDP as the chain transfer agent. MBMG monomer (0.3 g, 0.8 mmol), AIBN (0.4 mg, 2.6 μmol), and CDP (2.0 mg, 5.0 μmol) were dissolved in anhydrous DMSO. The initial monomer concentration for all polymerizations was 0.5 M, and trimesic acid (0.05M) was used as an internal ^1^H NMR standard. The polymerization mixtures were sparged for 30 min with high purity nitrogen gas. After sparging, the reaction vessel was placed into an oil bath at 70 °C and stirred. Aliquots were taken during the polymerization to track monomer conversion via ^1^H NMR spectroscopy. For the higher molecular weight targets, the amount of chain transfer agent was reduced to decrease the number of growing polymer chains while keeping the initial monomer concentration consistent. The polymerizations were quenched by exposing the reactions to air and cooling them in liquid nitrogen. The polymers were precipitated into excess water, isolated, and then lyophilized to dry the poly­(MBMG) (PMBMG). If no polymerization occurs, then no solid precipitates into the excess water; only after conversion to polymer is solid observed. The protected polymer was dissolved in methanol and an equal volume of HCl was added followed by stirring at RT for 3 h. The deprotected polymer was precipitated from the mixture using an excess of acetone at 40 °C and characterized by ^1^H NMR. Precipitation into excess acetone removes any excess monomer which is confirmed by the lack of vinyl groups in ^1^H NMR (Figure 1). The deprotected polymer is poly­(methyl-6-methacryloyl-β-d-glucosaminoside) (PMMG) (47% yield).

**Figure 1:**
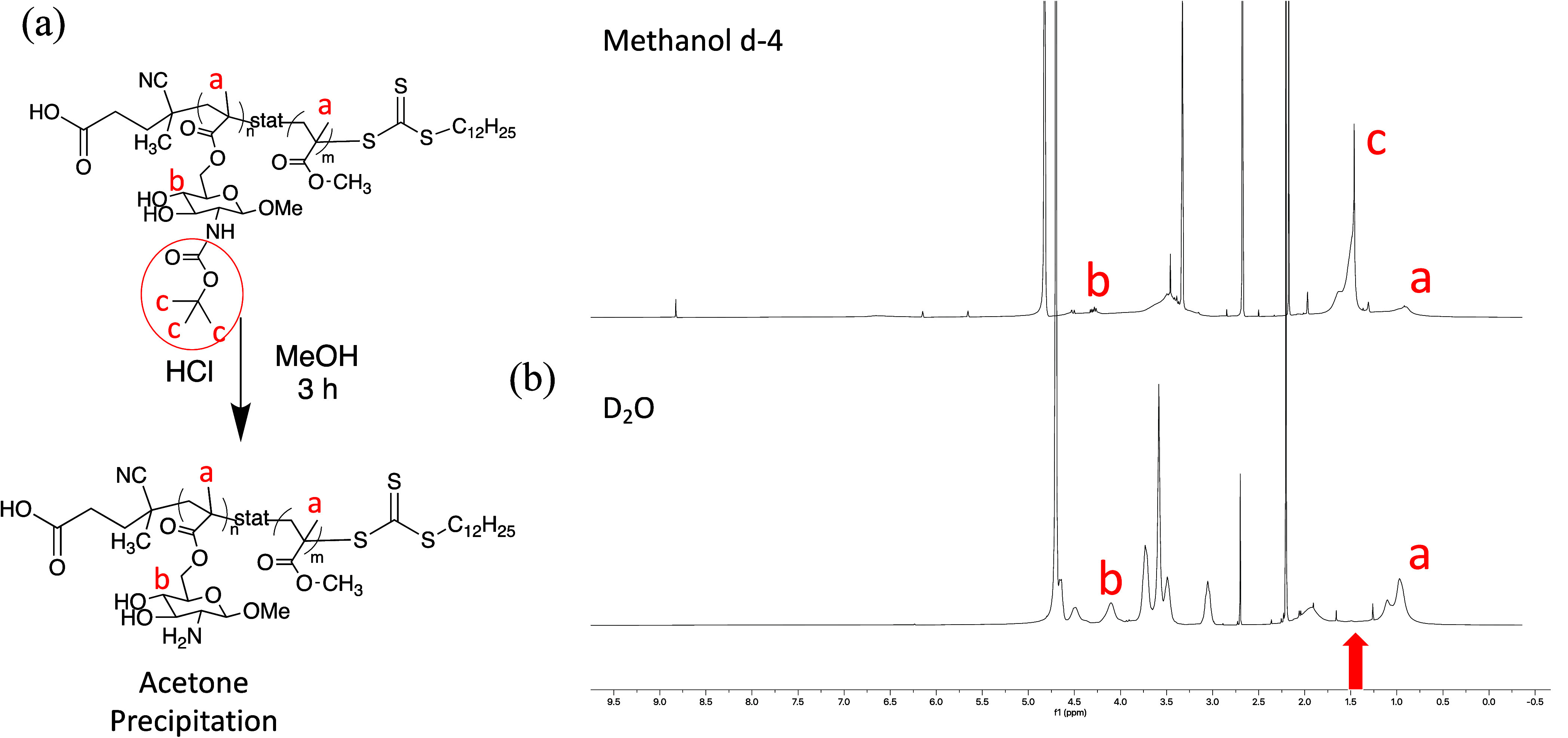
(a) Deprotection scheme of chitosan mimics after RAFT polymerization and (b) ^1^H NMR spectrum of the protected (top spectrum) and deprotected (bottom spectrum) polymers, showing disappearance of t-boc methyl groups.

#### Copolymerization

Low and high molecular weight copolymer chitosan mimics were prepared, and a typical polymerization utilized MBMG (0.5 g, 1.36 mmol) and MMA (0.046 mL, 0.34 mmol). The monomer concentration, thermal initiator, chain transfer agent, polymerization conditions, deprotection, and isolation were the same as those used in homopolymerization (42% yield). The molecular weight of the polymers was controlled by adjusting the concentration of CDP in the reactions, and the conversion of each monomer was tracked via ^1^H NMR spectroscopy.

#### Film Fabrication

Chitosan mimic films were prepared by dissolving the homopolymer or copolymer (10% w/w) in acetic acid (1% v/v aqueous solution), graphene oxide was added at 5 wt % of the polymer, and the mixture was shaken using a wrist-action shaker for 24 h. The polymer solutions were drop-cast onto tempered glass sheets to form nanocomposite films. Chitosan films for evaluation of amide levels in biodervied chitosan were prepared by drop casting from a 3 wt % chitosan in a 1% acetic solution. Bioderived chitosan nanocomposite films were fabricated by creating 2 wt % solutions of chitosan in 1% acetic acid aqueous solution, the graphene oxide was added at 1 wt % of the chitosan, and the mixtures were homogenized on a wrist-action shaker for 24 h. The films were formed using a draw-down bar with a wet thickness of ∼ 1 mm and allowed to dry at ambient temperatures.

#### NMR Spectroscopy

^1^H and ^13^C NMR spectroscopy was performed using a 600 MHz Bruker Advance III (TopSpin 3.1) spectrometer. Monomer and polymer spectra were acquired utilizing a delay time of 5 and 1 s, respectively. Monomer conversion was monitored by comparing the integrations of the vinyl peaks (5.0–7.0 ppm) to the unique proton peak of the trimesic acid (8.60 ppm) internal standard. Copolymer composition was determined by comparing integrations of the relative intensities of the saccharide anomeric proton on C1 (4.1 ppm) to the methyl protons of methyl methacrylate and MBMG (0.6–1.2 ppm). All NMR spectra were processed and analyzed using MestReNova software.

#### Aqueous Size Exclusion Chromatography with Multi-angle Laser Light Scattering (ASEC-MALLS)

Polymers were characterized using aqueous size exclusion chromatography (SEC) with multiangle laser light scattering on an Agilent 1260 Infinity II LC system with a Wyatt DAWN HELEOS-II light scattering detector (λ = 633 nm) and an Optilab T-rEX refractometer. An aqueous eluent of 0.1 M pH 4 acetate buffer at a flow rate of 0.25 mL/min at 25 °C was used with Eprogen Inc. CATSEC columns in series (1000, 300, and 100 Å). Values for polymer refractive index increment (d*n*/d*c*) were determined using an offline refractometer at 25 °C in 0.1 M pH 4 acetate buffer. Wyatt ASTRA SEC/LS software (version 7.1.4.8) was used to determine number-average molecular weight (*M*_n_), weight-average molecular weight (*M*_w_) and polymer dispersity (*Đ*).

#### Cytotoxicity

Human embryonic kidney cells (HEK293) were grown to 90% confluence in tissue culture polystyrene flasks in an incubator held at 37 °C and 5% CO_2_ using Dulbecco’s Modified Eagle Medium supplemented with 10% FBS and 1% penicillin-streptomycin. Trypsin was added to dissociate cells, and they were collected into a falcon tube and centrifuged at 5000 rpm for 5 min. HEK293 cells were resuspended in supplemented DMEM, and cells were counted using a hemocytometer to determine cell concentration. Cells were diluted with additional media to a working concentration of 6 × 10^4^ cells/mL and cells were seeded in a 96 well plate (200 μL volume, 12,000 cells/well). Seeded cells were left to adhere for 24 h in an incubator at 37 °C and 5% CO_2_. Each of the polymers was dissolved in 0.1% acetic acid solution at a concentration of 1 mg/mL. After 24 h, polymer solutions were added to the wells to obtain the desired concentration, with nuclease free water used as a negative control and Triton X-100 as a positive control for 100% cytotoxicity (i.e., complete LDH release). Cells were incubated for a further 24 h at 37 °C and 5% CO_2_ with treatments. Cytotoxicity was evaluated using the CyQUANT LDH Kit (Invitrogen) following manufacturer protocols and was calculated using eq 1. A microplate reader was used to assess the absorbance at 490 nm with a reference wavelength of 690 nm. The percent cytotoxicity was calculated as follows:

$$\%\;\mathrm{cytotoxicity}=\frac{A_{\mathrm{LDH,treated}}-A_{\mathrm{LDH,spont}}}{A_{\mathrm{LDH,max}}-A_{\mathrm{LDH,spont}}}\times 100\%$$

where *A*_LDH,treated_, *A*_LDH,spont_, and *A*_LDH,max_ are the compound-treated, spontaneous, and maximum LDH activities, respectively. The LDH activity was calculated as follows:

$$\mathrm{LDH}\;\mathrm{activity}=\frac{{\mathrm{Abs}}_{\mathrm{sample},490}-{\mathrm{Abs}}_{\mathrm{sample},690}}{{\mathrm{Abs}}_{\mathrm{MLDH},490}-{\mathrm{Abs}}_{\mathrm{MLDH},690}}\times 100\%$$

where Abs_S,λ_ is the absorbance of substance S at wavelength λ nm.

#### Hemolysis Assay

Triton X-100 was diluted to obtain 10% stock solutions by weight (w/v). The test compounds dissolved in PBS (pH 7.4) to obtain a 2% stock solution. The cytotoxicity of the compounds was determined following the protocol adopted from Sæbø et al.^32^ Briefly, blood samples were centrifuged at 1700X g for 5 min. The supernatant was removed by aspiration, and 2 mL of PBS (pH 7.4) was added. The washing step was repeated three times or until the supernatant was clear. After the final aspiration, the remaining pellet was diluted 1:100 in PBS pH 7 to prepare a 1% erythrocyte suspension. Using a 96-well plate, 50 μL of test compounds (LMW chitosan, HHP, and LCP), PBS (negative control), and 10% Triton X-100 (positive control) were mixed with 50 μL of blood sample (1% erythrocyte suspension). The samples were then incubated at 37 °C for 60 min. After incubation, the plates were centrifuged at 1700× g for 5 min, and 50 μL of the supernatants were transferred to transparent, flat-bottom 96-well plates. Finally, absorption was measured at 405 nm using a Victor Nivo Multimode microplate reader (PerkinElmer). All experimental results were analyzed using GraphPad Prism to generate figures and plots and to calculate statistics.

#### Ethics Statement

All animal procedures were conducted in strict accordance with the recommendations of the Guide for the Care and Use of Laboratory Animals of the National Institutes of Health, USA. The protocol for hamster blood was approved by the Institutional Animal Care and Use Committee (IACUC) of the University of Southern Mississippi (protocol # 17101206.5). All efforts were made to minimize animal distress and ensure their well-being throughout the study.

#### Minimum Inhibitory Concentration (MIC)

*E. coli* (strain: DH5a) and *S. aureus* (strain: RN4220) were recovered from a frozen stock, streaked onto a fresh Luria–Bertani (LB) agar plate, and grown overnight at 37 °C. A single colony was picked from the streaked plate and grown overnight in LB media at 37 °C on an orbital shaker (OHAUS) at 160 rpm. Bacteria from the overnight culture were added to fresh LB media and shaken at 37 °C for 2.5 h for the bacteria to reach the log phase. The log phase culture was diluted to a working concentration of ∼ 1 × 10^8^ CFU/mL. Chitosan and the synthetic mimics were dissolved in 0.5% acetic acid at 5 mg/mL and serially diluted to polymer concentrations between 0.0012 and 1.3 mg/mL with LB media. In a 96-well plate, each sample (75 μL) was loaded in quadruplicate along with wells loaded solely with the buffer to serve as a control. Subsequently, 25 μL of diluted bacterial culture was introduced into each well. Each plate was sealed with Parafilm and stored in a 37 °C incubator with shaking at 160 rpm overnight. Postincubation, optical density measurements at 600 nm were taken for each well. Significance between all samples was determined using a two-way *t* test and *p* < 0.05.

#### X-ray Photoelectron Spectroscopy (XPS)

XPS spectra were obtained from a Thermo-Fisher ESCALAB Xi+ spectrometer equipped with a monochromatic Al X-ray source (1486.6 eV). The graphene oxide powder or film samples were deposited onto double sided copper tape attached to the sample holder. Measurements were performed using the standard magnetic lens mode and charge compensation. The base pressure in the analysis chamber during spectral acquisition was 3 × 10–7 mbar. The pass energy of the analyzer was set at 20 eV for high-resolution spectra and 150 eV for survey scans with energy resolutions of 0.1 and 1.0 eV, respectively. Binding energies were calibrated with respect to C 1s at 284.8 eV. All spectra were recorded using the Thermo Scientific Avantage software; data files were translated to VGD format and processed using the Thermo Avantage package v5.9904. Significance between all samples was determined using a two-way *t* test and *p* < 0.05.

#### Wide Angle X-ray Scattering (WAXS)

A Xeuss 2.0 laboratory beamline (Xenocs Inc.) with an X-ray wavelength of 0.154 nm and sample-to-detector distance of 2.5 m was used to perform WAXS and SAXS. Films were folded to increase thickness to ∼ 0.1 mm, and data was processed with IgorPro software.

#### Tensile Testing

ASTM type V tensile bars were cut from the chitosan/graphene oxide films, and the tensile properties were evaluated using a TA RSA-G2 Solids Analyzer in tension mode. The gap used for all experiments was 10 mm, and the elongation rate was set at 0.1 mm/s with a sampling rate of 10 pts/s. Samples were tested at 25 °C. Film thicknesses were measured using a Bruker Dektak XT stylus profilometer. The stylus force was 1 mg with a height range of 524 μm and a measurement length of 2 mm. The measurement mode was hills and valley with a scan time of 60s. All film thicknesses were measured in triplicate. Significance between all samples was determined using a two-way *t* test and *p* < 0.05.

## Results and Discussion

### Synthesis of Glycomonomer

MBMG was synthesized through a five-step process starting with glucosamine-HCl (Scheme 1).

**Scheme 1:**
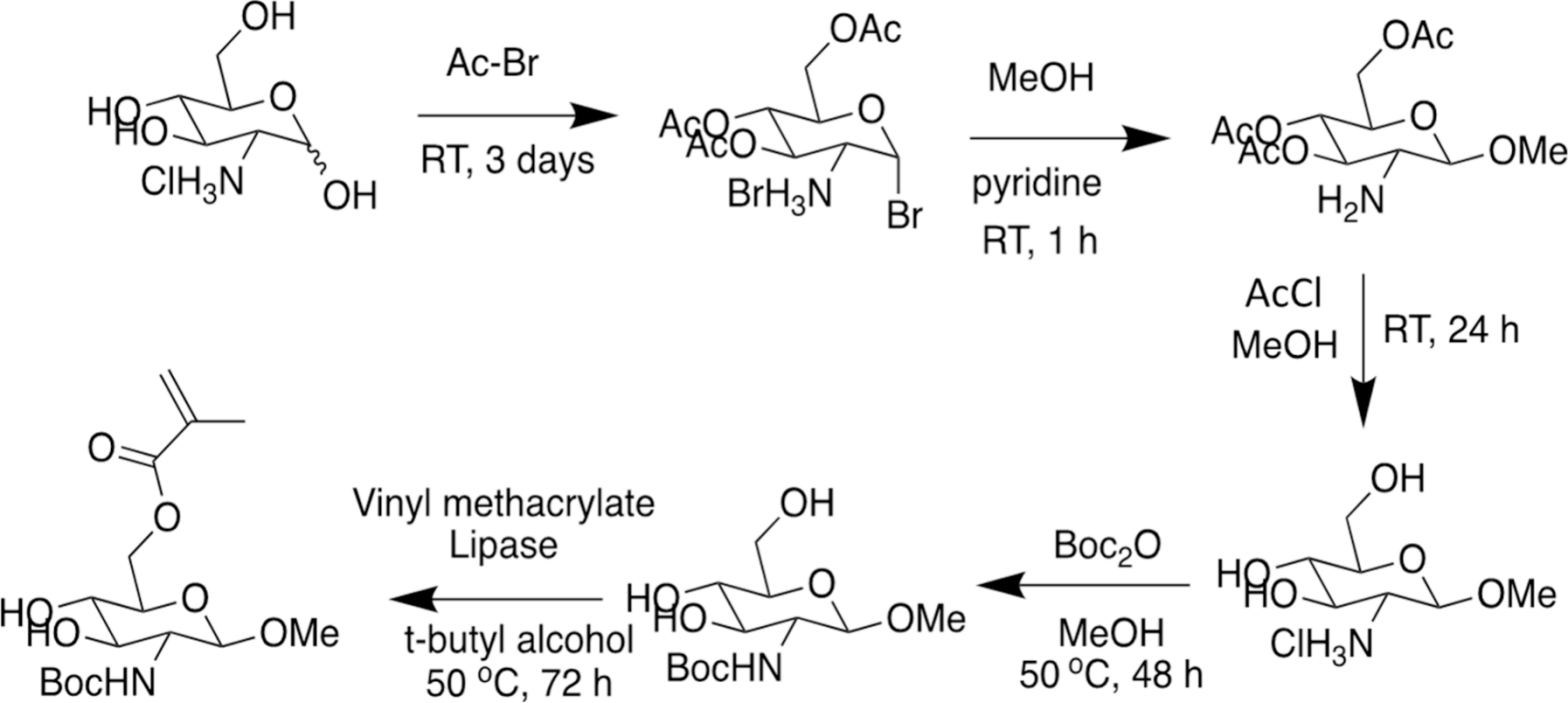
Synthesis of Methyl *N*-Boc-6-methacryloyl-β-d-glucosaminoside Glycomonomer

The first three synthetic steps were conducted according to a procedure by Liau et al. and Billing et al. without modification, and the product, methyl glucosaminoside-HCl, was characterized via ^1^H NMR spectroscopy (Figure S1, 53% combined yield).^13,31^ This structure with the methoxy group at C1 was used to mimic the glycosidic linkage between monomer units of chitosan. The primary amine of methyl glucosaminoside-HCl was protected with tert-butyloxycarbonyl (t-boc) and confirmed by ^1^H NMR spectroscopy (Figure S2, 60% yield). Protecting the primary amine with a t-boc group limits side reactions during the final step of the monomer synthesis and eliminates the potential for Michael addition during polymerization. The final step of the monomer synthesis was the regioselective addition of the methacrylate group at the C6 position. Several esterification methods were attempted following literature procedures for cellulose that utilized methacryloyl chloride or methacrylic anhydride.^3,13,33,34^ These methods resulted in the esterification of not only the C6 hydroxyl but also the hydroxyls at the C3 and C4 positions. Adapting procedures used for the modification of glucose, MBMG was produced using lipase-mediated transesterification with vinyl methacrylate.^35,36^ The resulting MBMG glycomonomer was purified using column chromatography and characterized using ^1^H and ^13^C NMR spectroscopy (35% yield) (Figure S3).

### RAFT Polymerizations of MBMG Homopolymer and Copolymer Chitosan Mimics

Most commercial chitosan polymers are between 75 and 95% deacetylated, and to mimic this variability, homopolymers of MBMG (representing fully deacetylated chitosan) and copolymers with 20 mol % MMA (representing partially deacetylated chitosan) were synthesized using RAFT polymerization (Scheme 2).

**Scheme 2:**
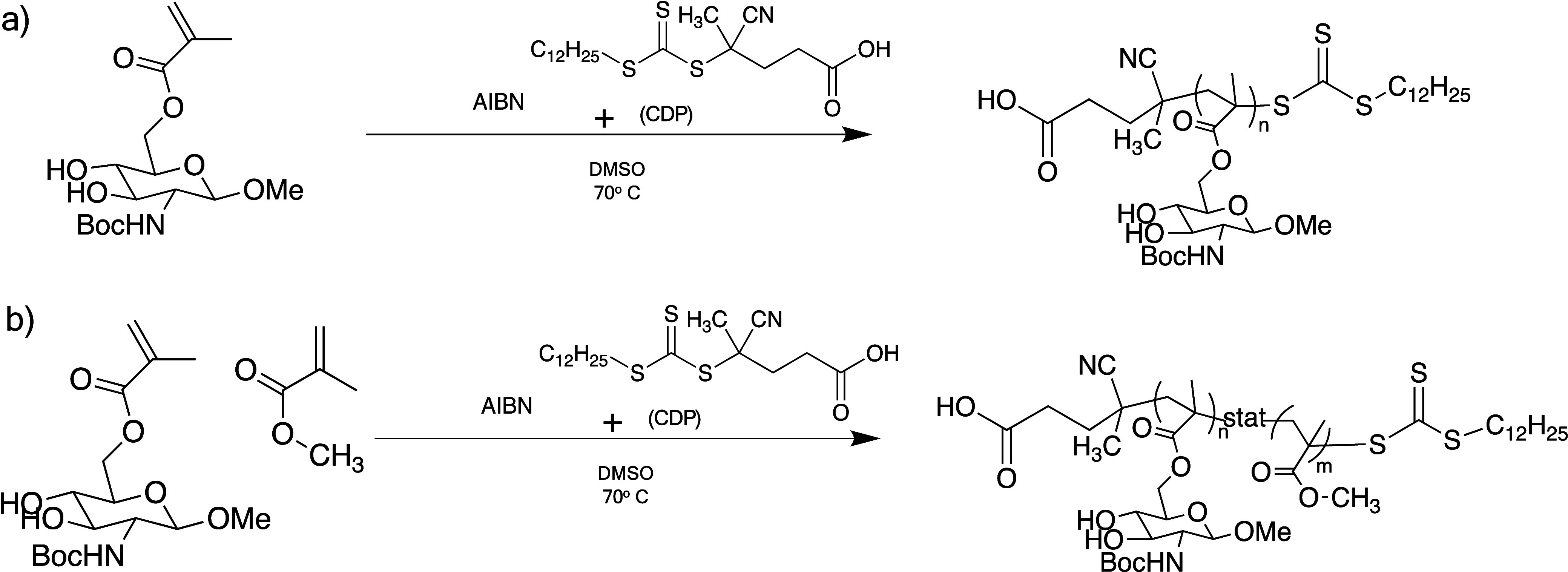
RAFT Polymerization of Methyl *N*-Boc-6-methacryloyl-β-d-glucosaminoside as a) a Homopolymer and b) a Copolymer with MMA

RAFT polymerizations were originally attempted using 4-cyano-4-(phenylcarbonothioylthio) pentanoic acid as the CTA based on a procedure by Albertin et al.,^35^ but due to poor stability during the polymerization, we elected to use the more stable trithiol CDP. Two homopolymers of low (LHP) and high (HHP) molecular weights and two copolymers of low (LCP) and high (HCP) molecular weights were synthesized (Table 1).

**Table 1:** Molecular Weights, Compositions, and Dispersity of Synthetic Chitosan Mimics

				mol % MMA	
sample	*M*_w_ (kDa)^a^	*M*_n_ (kDa)^a^	*Đ*	feed	copolymer^b^
LHP	94	93	1.01		
HHP	165	162	1.02		
LCP	57	56	1.02	20	24
HCP	124	120	1.03	20	21

a Determined using ASEC-MALLS in 0.1 M sodium acetate (pH 4) with Eprogen CATSEC columns in series (1000, 300, and 100 Å).

b Copolymer composition determined by comparing the integrations of the peaks for the C1 proton of the glycomonomer unit (δ 4.1, 1H) and the methyl group of the polymer backbone (δ 1.0, 3H).

All polymerizations were performed with the t-boc protecting group on the monomer followed by deprotection. The t-boc protecting groups were removed using strong acid (HCl) and confirmed via ^1^H NMR spectroscopy (Figure 1).

RAFT polymerization provided the appropriate control to synthesize both homopolymers and copolymers with low dispersities and targeted molecular weights (Table 1), with monomodal narrow peaks observed in SEC-MALLS traces (Figure S4). Copolymerization was performed with a monomer ratio of 80 mol % MBMG and 20 mol % MMA, and monomer incorporation was determined by ^1^H NMR (Figure 2). MMA incorporation in the copolymer is close to the reaction feed of 20% MMA. Production of chitosan from chitin is a multistep process, making molecular weight and degree of deacetylation difficult to control. The utilization of RAFT polymerization allowed for the synthesis of mimics that maintained the primary structural features of the repeat units of chitosan while also controlling molecular weight, dispersity, and copolymer structure. The mimics incorporate the same main glucosamine unit, which has a primary amine that is similar to that of chitosan. The primary amine of chitosan is located at the 2 position on the sugar with a hydroxyl unit at the 3 position and a glycosidic linkage at the 1 position; our mimic has the primary amine at the 2 position and a hydroxyl at the 3 position, and we installed a methoxy group to mimic the glycosidic linkage and keep the ring from opening. Unlike chitosan, the glucosamine units are not a part of the polymer backbone but instead are pendant units from the methacrylate backbone, allowing us to create well-defined polymers with the advantage of water solubility while retaining the primary functionalities of chitosan. The structural similarity of the glucosamine unit allows both the comparison of nanoparticle interactions of chitosan and the mimics, and the exploration of material properties of the mimics, including cytotoxicity, hemolysis, and antimicrobial properties, for potential suitability in biomaterials applications, such as delivery vehicles for therapeutics.

**Figure 2:**
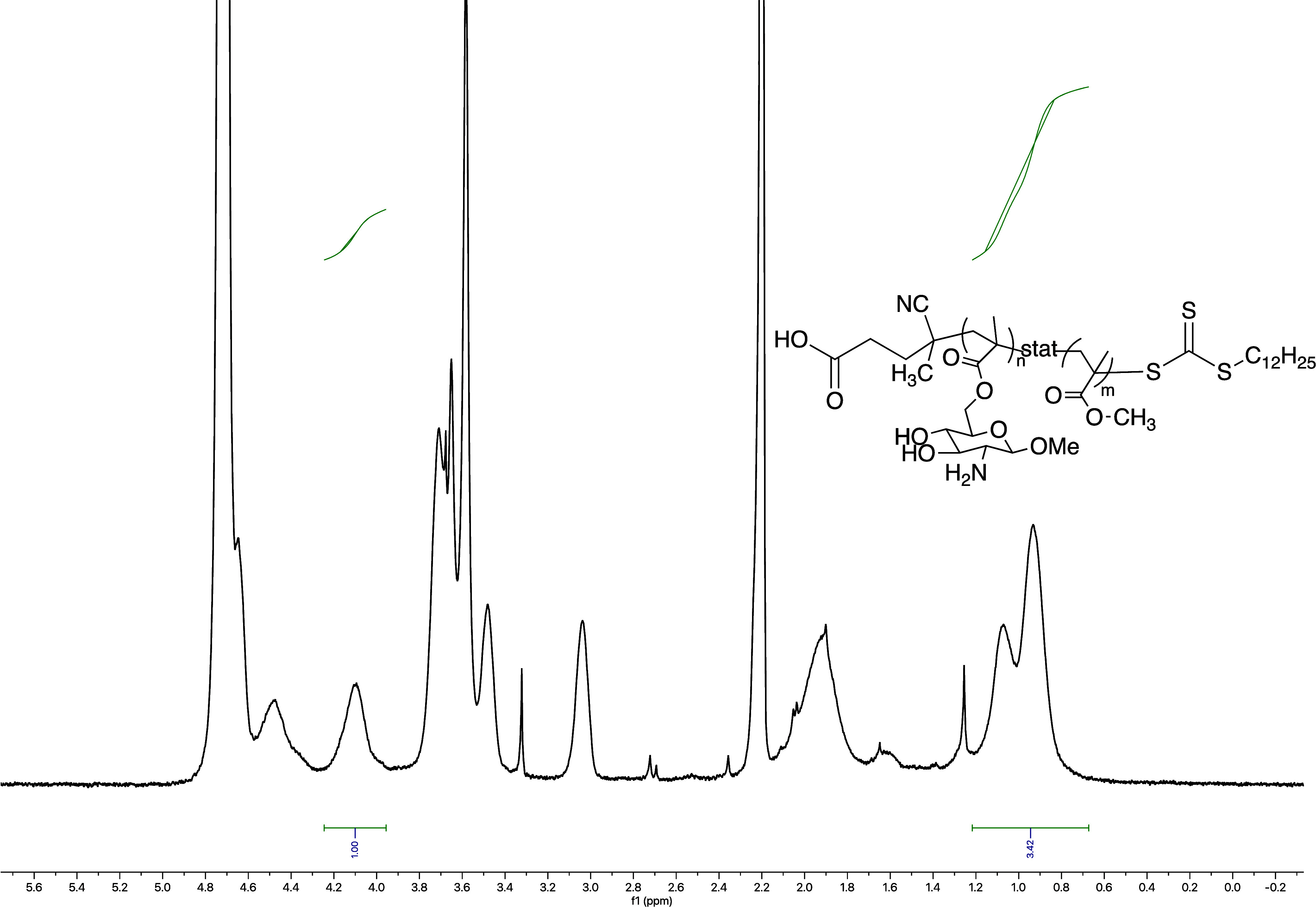
^1^H NMR analysis was used to determine the incorporation of MMA in the copolymer mimics of chitosan. Copolymer incorporation was determined using the integration value of the C1 proton (1H) (δ 4.1 ppm) of the glycomonomer units compared to the combined integration value of the methyl groups (6H) (δ1.2–0.7 ppm) along the backbone of the polymer.

### Cytotoxicity

LDH assays were performed with HEK293 cells to investigate the cytotoxicity of the synthetic mimics compared to low molecular weight chitosan (Figure 3).

**Figure 3:**
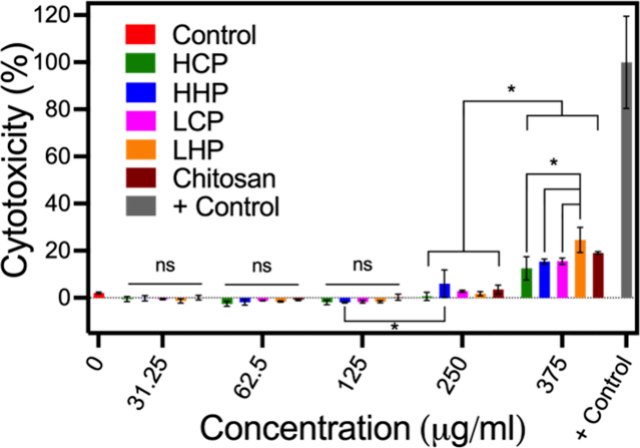
Evaluation of cytotoxicity of the synthetic glycopolymer chitosan mimics compared to chitosan at 24 h. Negative control is nuclease-free water and positive control is Triton X-100 (+ Control). No significant difference is observed between chitosan and synthetic glycopolymers up to 375 μg/mL. At 375 μg/mL, each of the mimics and chitosan become significantly more toxic, but there is no significant difference between chitosan and the mimics. Data displayed as mean ± standard deviation (*n* = 3 per treatment), with significance assessed with a one-way analysis of variance (ANOVA) and a posthoc Tukey test (*p < 0.05).

Chitosan and each of the mimics had relatively low cytotoxicity (<10%) up to 250 μg/mL, and no significant difference between chitosan and the mimics was observed. At 375 μg/mL, toxicity was significantly increased for all systems, with no significant difference between chitosan and the mimics. Two other significant changes were observed: HHP increased slightly in toxicity from 125 to 250 μg/mL (p = 0.0448), and LHP was more toxic than the other mimics at 375 μg/mL, but not more toxic than chitosan. However, these changes are not large (all systems show toxicity less than 10% at 250 μg/mL and less than 25% at 375 μg/mL), and no mimic shows significantly increased toxicity when compared to chitosan. Thus, the synthesized chitosan mimics have a greater degree of control over molecular weight and degree of deacetylation without enhancing the toxicity. Further, Further, the values measured for chitosan cytotoxicity are in good agreement with previous reports of chitosan with similar molecular weight and degree of deacetylation at the same concentrations.^37,38^ Thus, our synthetic polymers have similar compatibility with mammalian cells as chitosan with added versatility due to their solubility in water independent of the solution pH.

### Hemolysis Studies

The hemolytic effects of LCP, HHP, and low molecular weight chitosan in comparison to PBS (negative control) and Triton X-100 (positive control) were evaluated using blood from Syrian hamsters as described in the experimental section. Samples were incubated with 1% washed erythrocytes for 60 min at 37 °C, after which OD was measured to determine the amount of hemoglobin released (Figure 4). Chitosan (82.2%) and the positive control (92.6%) exhibit a significant destructive effect on the erythrocytes with a high degree of hemolysis. No significant difference is observed between the negative control (14.9%), HHP (16.8%), and LCP (14.7%), indicating a low degree of hemolysis.

**Figure 4:**
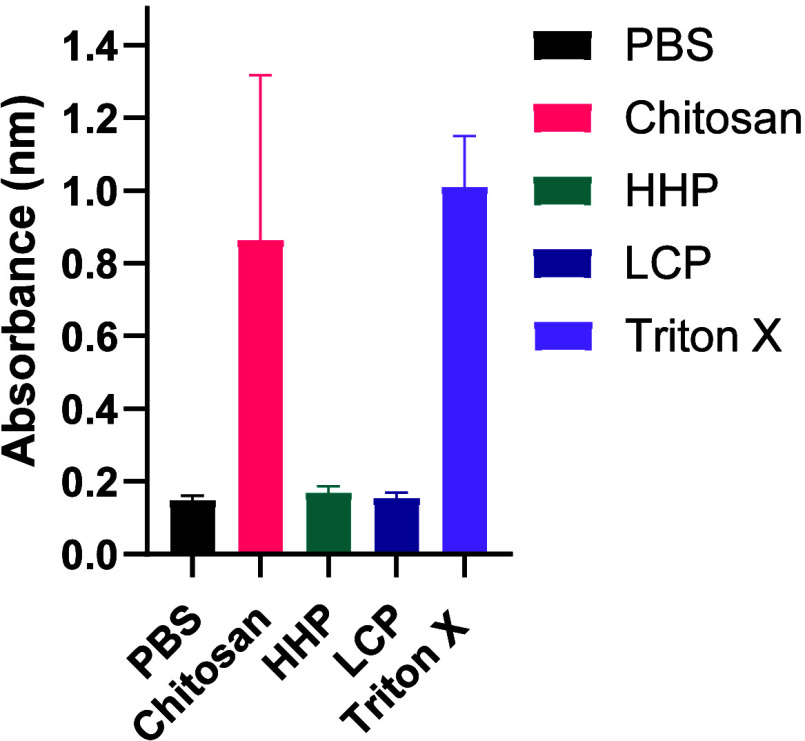
OD measurements of free hemoglobin in a 1% erythrocyte solution from Syrian hamsters at 405 nm (*Y*-axis) after they were incubated for 60 min at 37 °C with PBS (negative control), 10% Triton X-100 (positive control), LMW chitosan, HHP, and LCP as the test compounds. Error bars (SD) are plotted alongside the average values from three experimental replicates, each of which has two technical replicates. Chitosan and the positive control show a high degree of hemolysis, while the negative control, HHP, and LCP show a low degree of hemolysis.

### Bacterial Minimum Inhibitory Concentration (MIC)

The growth of Gram-negative *E. coli* and Gram-positive *S. aureus* bacteria in the presence of HHP and HCP were compared to that of the low molecular weight chitosan, and the optical density as a function of concentration is provided in Figures 5a and 5b. MIC, defined as the average concentration needed to achieve an optical density <0.2, was determined and is plotted in Figure 5c.

**Figure 5:**
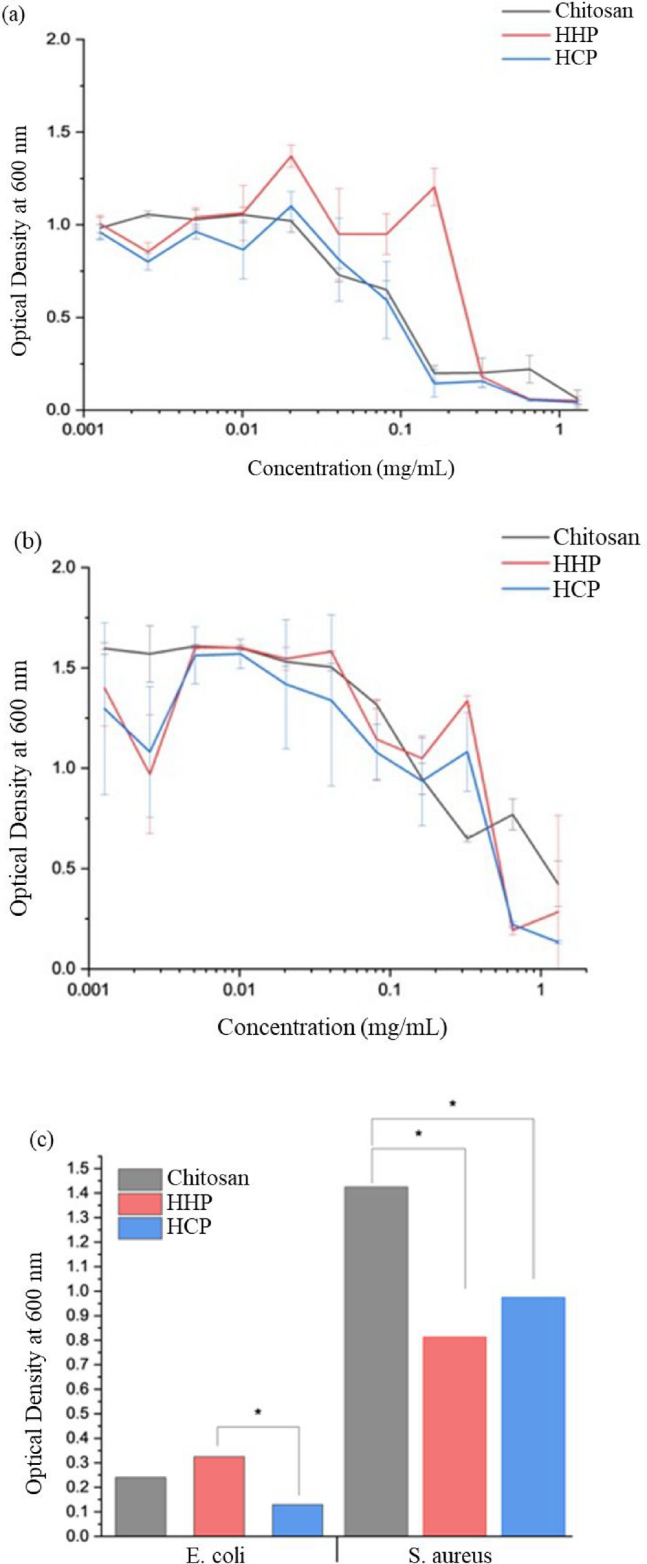
Similar bacterial growth inhibition was observed for chitosan and chitosan mimics for (a) *E. coli* (b) *S. aureus*. (c) The minimum inhibitory concentration for each polymer is defined as OD at 600 nm <0.2. Both HHP and HCP show significantly lower MIC for *S. aureus* than chitosan but show no significant improvement in inhibition for *E. coli* in comparison to chitosan (*P* < 0.05).

The HCP shows an *E. coli* growth profile similar to that of chitosan, with inhibition beginning at 0.04 mg/mL, while the HHP requires higher concentrations. The MICs for the three samples are similar, with HHP showing a higher value than HCP, but there is no significant difference between the mimics and chitosan. There is greater variability in the Gram-positive *S. aureus* growth profiles, and higher concentrations are required to inhibit growth for all three samples. In this case, both chitosan mimics provided statistically significant improvement in MIC in comparison to chitosan. Study of the antibacterial performance of chitosan is complicated by its limited solubility. Chitosan must be dissolved in acetic acid, which is known to have antimicrobial activity. The mimics were dissolved in acetic acid of the same concentration as chitosan to allow direct comparison of the antibacterial activity. All solutions were diluted to below the 0.2% (v/v) acetic acid concentration determined to be antibacterial.^13^ Our measured MIC values for chitosan are consistent with previous literature reports.^39,40^ The antibacterial performance of the synthetic glycopolymers is similar to or better than that of chitosan over the concentration ranges studied, and their improved solubility in water may create new applications in medical and personal care products.

### Chitosan Mimics and Graphene Oxide Interactions

Well characterized chitosan mimics of target molecular weight, low dispersity, and known composition provide models for study of chitosan/graphene oxide interactions. The primary goal of synthesizing the mimics was to study the interactions between the primary amine units and the functionalities of GO. Using a fractional factorial statistical design of experiments (DOE), 8 combinations of mimics with GO were targeted to evaluate the impact of four factors (GO size, GO functionality, mimic *M*_w_, and mimic composition) on CH/GO interactions (Table S1). Graphene oxide samples with lateral size ranges of 0.5–5 μm and 90–120 nm were modified using thermal reduction (300 °C) under N_2_. GO carbon/oxygen ratios before and after reduction were confirmed via deconvolution of XPS C 1s spectra via the peak fitting function (convergence <10^–4^) in Avantage software (Figure 6a-d), and the GO lateral size reported by the supplier was confirmed using TEM (Figure 6e,f). Table S2 provides the C/O ratios for the micron scale reduced (μm rGO) and unreduced (μm GO) samples and nanometer scale reduced (nm rGO) and unreduced (nm GO) samples.

**Figure 6:**
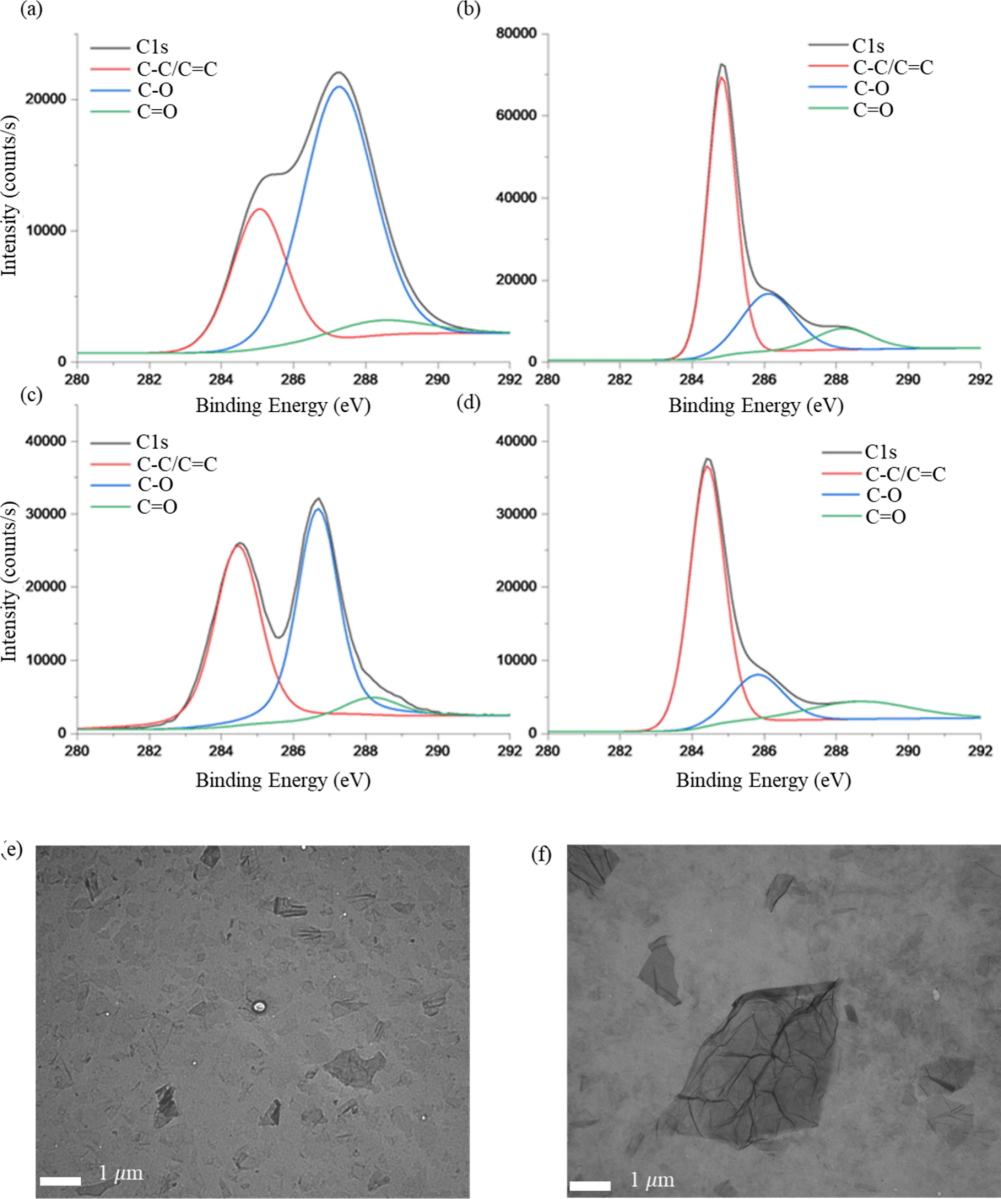
(a-d) C 1s XPS spectra of: (a) nanometer scale graphene oxide as received (b) and after thermal reduction; (c) micrometer scale graphene oxide as received (d) and after reduction. (e-f) TEM images of: (e) nanometer scale graphene oxide particles (f) micrometer scale graphene oxide particles.

For both systems, the primary change in the spectra after reduction is a decrease in the C–O peak at 286 eV, indicating that epoxides were the major functional groups removed. Each nanocomposite film contained 5 wt % GO, and the bonding between the primary amine of the mimics and GO was evaluated via XPS. Careful design of the mimics allowed for the evaluation of covalent bonding between the primary amine and GO without the complication of the amide functionality present in the N-acetylglucosamine units of chitosan (Figure 7a). XPS can be used to differentiate between types of nitrogen using deconvolution of the N 1s scan. Three peaks are common for nitrogen groups in chitosan samples: 398 eV (primary amine), 400.5 eV (amide), and 401.7 eV (protonated amine) (Figure 7b).^16,41^ The films made with chitosan mimics and GO had only two nitrogen peaks present at 398 and 401.7 eV, representing the primary amine and protonated amine, respectively (Figure 7c). The lack of emergence of an amide peak indicates that no amidation reaction occurred between the primary amine of the mimic and graphene oxide during the film formation process. No significant difference in protonation of the primary amine was observed for any of the mimic formulations evaluated in the DOE, shown in the analysis of variance (ANOVA, Table S3). It is well-known that natural polysaccharides, including chitosan, display wide variation in their structures.^42^ Because no amide formation and no significant difference in primary amine protonation was found in the DOE analysis of the mimics, we explored the natural variability in the types of nitrogen present in our commercial chitosan sample. Five films from a single chitosan solution were prepared via drop casting, and XPS was utilized to measure the range of amidation (found to be 7–12%) and protonated amine (found to be 8–15%), with results shown in Table S4. This high level of natural variation demonstrates the difficulty in quantifying the interactions between chitosan amine and amide groups and GO, and the benefit of the creation of well-defined chitosan mimics to model the interactions. As the mimics do not contain amide functionality, the appearance of amide peaks on blending with GO would indicate the formation of new covalent bonds between the primary amine and GO. No peaks were observed at 400.5 eV for any of the mimic films, allowing us to infer that under these common film forming conditions, no amidation occurs between chitosan and GO. The lack of amidation reaction and lack of significant differences in protonation of the amine in the mimics indicate that differences in properties of nanocomposites with different GO size and functionality are independent of amine interactions. It is likely that nanocomposite properties are determined primarily by hydrogen bonding between GO and chitosan.^43,44^

**Figure 7:**
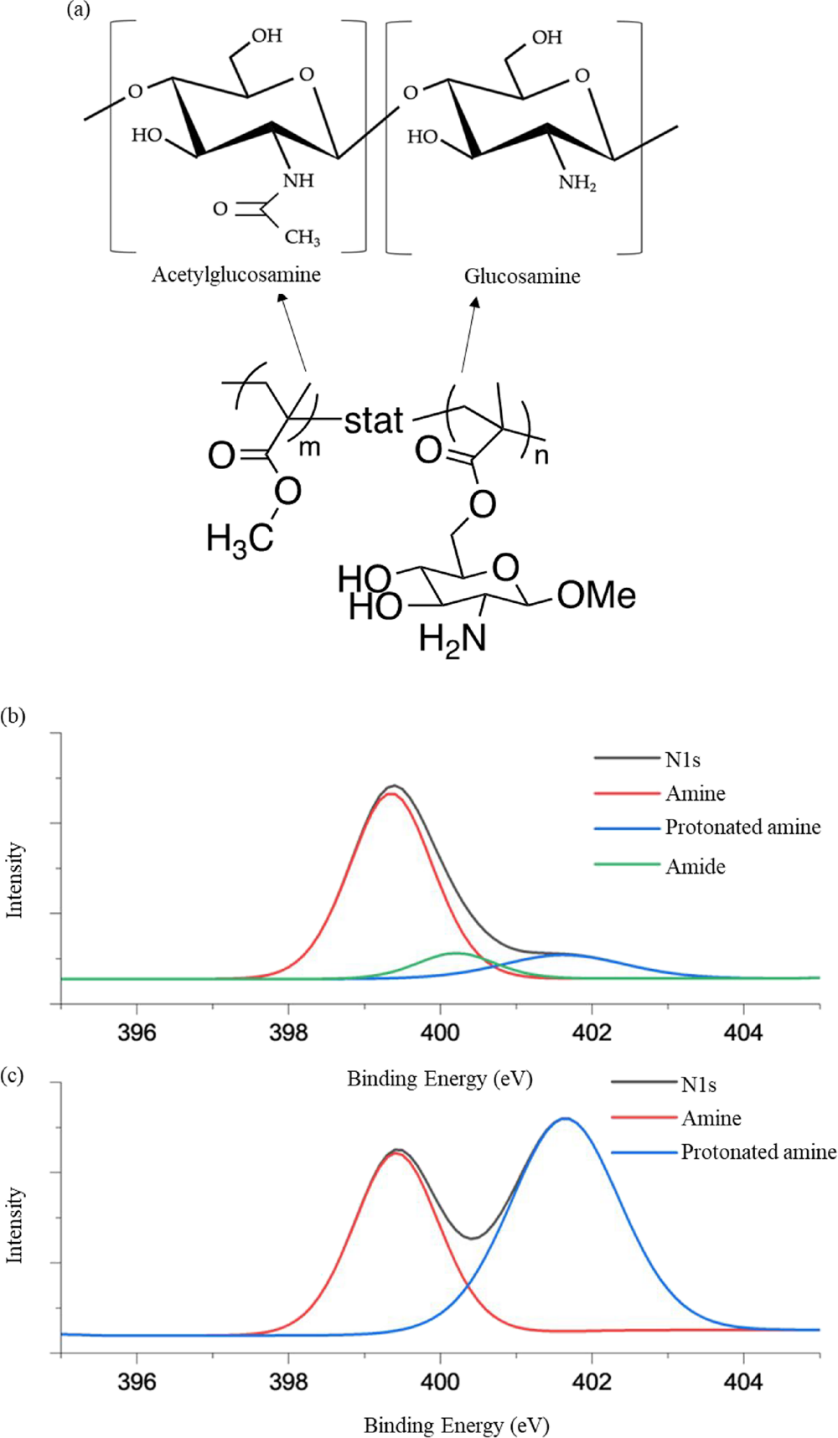
(a) Structure of chitosan and the copolymer designed to mimic the two saccharide units. XPS spectra of films containing 5% graphene oxide: (a) natural chitosan (b) HCP chitosan mimic (c).

### Chitosan/GO Films

The impact of GO additive size and level of oxygen functionality on chitosan film properties is of interest to better understand the design parameters for chitosan/GO composites. The same GO materials used for the mimic studies were blended in solution at 1 wt % of the medium molecular weight chitosan and fabricated into films as described in the methods section. XPS evaluations of the films are consistent with the results of the chitosan mimic evaluation, with no statistically significant differences observed in the amount of amidation or protonation with the addition of GO (Table 2, Figure S5) (two-way *t* tests, *P* < 0.05).

**Table 2:** Percentages of Primary Amines, Amides, and Protonated Amines from Deconvolution of N 1s Spectra X-ray Photoelectron Spectroscopy Measurements of Chitosan/GO Films

	Primary amine (399 eV)	Amide (400.5 eV)	Protonated amine (401.7 eV)
**Neat CH**	79±4%	10±2%	11±3%
**CH nm GO**	79±7%	10±2%	11±4%
**CH nm rGO**	82±1%	10±1%	8±1%
**CH μm GO**	80±6%	12±4%	8±5%
**CH μm rGO**	80±5%	10±3%	10±4%

Representative tensile curves of the five samples listed in Table 2 are shown in Figure 8a, and the values of ultimate tensile strength, elongation at break, and Young’s modulus are provided in Figure 8b. Our evaluation of interactions between the GO and chitosan showed no significant differences in electrostatic or covalent interactions between the primary amine and GO. All samples with the addition of GO show statistically significant increases in tensile strength and modulus compared to the neat chitosan. The increase in strength can be attributed to the strong hydrogen bonding and electrostatic interactions between GO and chitosan.^14,25^ The μm GO provides a greater increase in strength and modulus than the nm GO, but within the size category, no significant effect is observed for reduction of GO, indicating that size is of greater importance in determining strength than composition and functionality of GO. Nanometer GO increased the elongation at break compared to neat chitosan, while μm GOs decreased the elongation. Again, no significant differences were observed for reduced and unreduced GO within the size category. Average Young’s modulus increased for all samples containing GO. The increase in toughness with the nanometer scale additive is attributed to the disruption of hydrogen bonding between chitosan polymer chains, allowing more elongation prior to break under tension.^16^ WAXS was utilized to evaluate the impact of crystallinity on the observed tensile properties. The WAXS scattering patterns show only two peaks (15.3°, 20.5°), and no observed shift in the scattering pattern or the intensity of scattering is observed with the addition of GO of any size or carbon/oxygen ratio (Figure 9). The lack of change in crystallinity suggests that the changes in strength and toughness of the nanocomposite films are driven by the dispersion and hydrogen bonding interactions between chitosan and GO.

**Figure 8:**
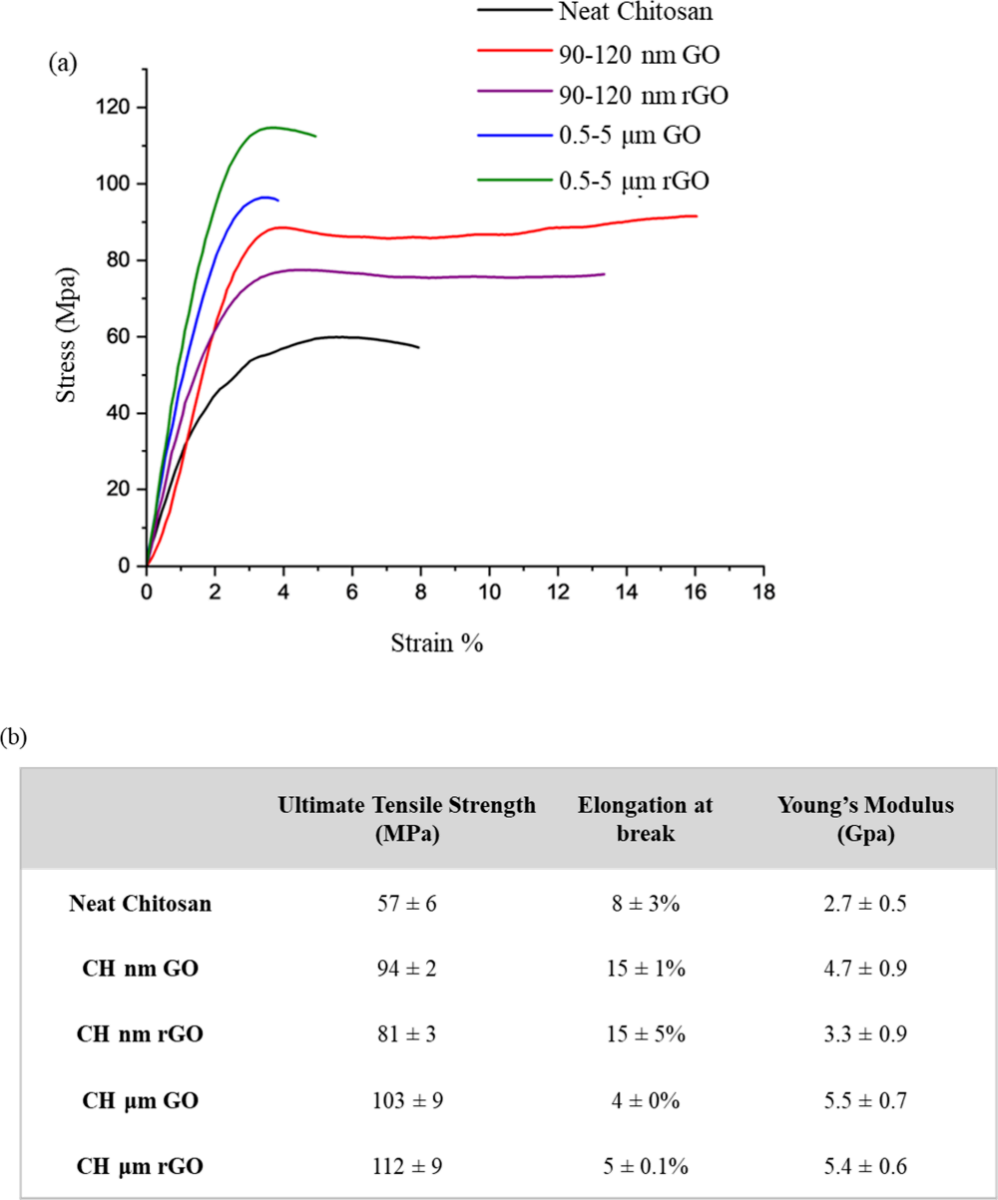
(a) Stress vs strain overlay of chitosan/GO nanocomposite films. (b) Table of ultimate tensile strength and elongation values with the average and one standard deviation of replicates.

**Figure 9:**
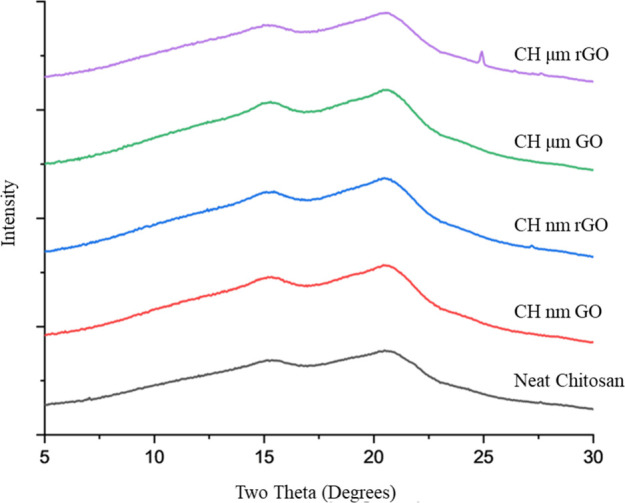
Wide angle X-ray scattering patterns of chitosan with graphene oxide samples showing no change in crystallinity with changes in GO additive.

## Conclusions

A new monomer was synthesized and used to make four new polymers as synthetic mimics of chitosan. The mimics have cytotoxicity and antibacterial activity similar to that of naturally derived chitosan polymer, with the advantages of having low dispersity and being fully water-soluble. Using the well-defined mimics, the interactions between typical chitosan functional groups and graphene oxide of various sizes and C/O ratios were probed, and no indication of covalent bond formation was observed. Lastly, the same modified graphene oxides were blended with bioderived chitosan to determine the impact on mechanical properties, and the addition of nanometer scale graphene oxide increased both the strength (>42% increase) and toughness (>85% increase) of chitosan films, while micron scale GO increased strength (>80% increase) but decreased the toughness (50% reduction). This work is a systematic evaluation of the structure/property relationships of well-defined chitosan mimics and GO with different physical and chemical properties, which provides context for understanding the wide variation in reported chitosan/GO nanocomposite properties. In addition, our chitosan mimics are antibacterial, biocompatible, and water-soluble with potential biomedical, environmental, and personal care applications.

## Supplementary Material



## Notes

NMR spectra of monomer precursors and monomer: methyl glucosaminoside-HCl, *N*-boc-β-d-glucosaminoside, and methyl *N*-boc-6-methacryloyl-β-d-glucosaminoside; SEC-MALLS traces of LHP, HHP, LCP, and HCP glycopolymer chitosan mimics; fractional factorial statistical design of experiments XPS data and ANOVA tables; table of graphene oxide particle size and ratios of carbon to oxygen before and after thermal reduction; table of amide and amine variation in commercial chitin sample; XPS spectra of chitosan/GO films (PDF)

## References

[1] Ruiz, G. A. M. ; Corrales, H. F. Z. , Chitosan, Chitosan Derivatives and their Biomedical Applications. In Biological Activities and Application of Marine Polysaccharides, 2017.

[2] Pillai C. K.
S., Paul W., Sharma C. P. (2009). Chitin and chitosan polymers: Chemistry,
solubility and fiber formation. Prog. Polym.
Sci..

[3] Mourya V. K., Inamdar N. N. (2008). Chitosan-modifications
and applications: Opportunities
galore. React. Funct. Polym..

[4] Kumari, S. ; Kishor, R. , Chitin and chitosan: origin, properties, and applications. In Handbook of Chitin and Chitosan, 2020; pp 1–33.

[5] Kumar S., Ye F., Dobretsov S., Dutta J. (2019). Chitosan Nanocomposite Coatings for
Food, Paints, and Water Treatment Applications. Applied Sciences.

[6] Kolodziejska M., Jankowska K., Klak M., Wszola M. (2021). Chitosan as an Underrated
Polymer in Modern Tissue Engineering. Nanomaterials
(Basel).

[7] Akpan, E. I. ; Gbenebor, O. P. ; Adeosun, S. O. ; Cletus, O. , Solubility, degree of acetylation, and distribution of acetyl groups in chitosan. In Handbook of Chitin and Chitosan, 2020; pp 131–164.

[8] Foster L. J., Ho S., Hook J., Basuki M., Marcal H. (2015). Chitosan as a Biomaterial:
Influence of Degree of Deacetylation on Its Physiochemical, Material
and Biological Properties. PLoS One.

[9] Li N., Qu X., Wang L., Tian Q., Chen Y., Yao X., Chen S., Jin S. (2020). Chemical synthesis of chitosan-mimetic
polymers via ring-opening metathesis polymerization and their applications
in Cu2+ adsorption and catalytic decomposition. Polym. Chem..

[10] Kadokawa J., Shimohigoshi R., Yamashita K., Yamamoto K. (2015). Synthesis of chitin
and chitosan stereoisomers by thermostable alpha-glucan phosphorylase-catalyzed
enzymatic polymerization of alpha-D-glucosamine 1-phosphate. Org. Biomol Chem..

[11] Yamada K. (2000). Minoda, Masahiko,
Miyamoto, Takeaki, Controlled Synthesis of Glycopolymers with Pendant
D- Glucosamine Residues by Living Cationic Polymerization. J. Polym. Chem.: Part A.

[12] Du A. W., Lu H., Stenzel M. H. (2017). Cationic glycopolymers through controlled polymerisation
of a glucosamine-based monomer mimicking the behaviour of chitosan. Polym. Chem..

[13] Liau W. T., Kasko A. M. (2017). Poly­(methyl 6-acryloyl-beta-d-glucosaminoside)
as a
Cationic Glycomimetic of Chitosan. Biomacromolecules.

[14] Han D., Yan L., Chen W., Li W. (2011). Preparation of chitosan/graphene
oxide composite film with enhanced mechanical strength in the wet
state. Carbohydr. Polym..

[15] Menazea A. A., Ezzat H. A., Omara W., Basyouni O. H., Ibrahim S. A., Mohamed A. A., Tawfik W., Ibrahim M. A. (2020). Chitosan/graphene
oxide composite as an effective removal of Ni, Cu, As, Cd and Pb from
wastewater. Comput. Theor. Chem..

[16] Abolhassani M., Griggs C. S., Gurtowski L. A., Mattei-Sosa J. A., Nevins M., Medina V. F., Morgan T. A., Greenlee L. F. (2017). Scalable
Chitosan-Graphene Oxide Membranes: The Effect of GO Size on Properties
and Cross-Flow Filtration Performance. ACS Omega.

[17] Sabzevari M., Cree D. E., Wilson L. D. (2018). Graphene Oxide-Chitosan Composite
Material for Treatment of a Model Dye Effluent. ACS Omega.

[18] Wang J., Gao X., Wang J., Wei Y., Li Z., Gao C. (2015). O-(carboxymethyl)-chitosan
nanofiltration membrane surface functionalized with graphene oxide
nanosheets for enhanced desalting properties. ACS Appl. Mater. Interfaces.

[19] Singh A., Sinsinbar G., Choudhary M., Kumar V., Pasricha R., Verma H. N., Singh S. P., Arora K. (2013). Graphene oxide-chitosan
nanocomposite based electrochemical DNA biosensor for detection of
typhoid. Sens. Actuators, B.

[20] He L., Wang H., Xia G., Sun J., Song R. (2014). Chitosan/graphene
oxide nanocomposite films with enhanced interfacial interaction and
their electrochemical applications. Appl. Surf.
Sci..

[21] Begum H., Ahmed M. S., Jeon S. (2017). New Approach for Porous Chitosan-Graphene
Matrix Preparation through Enhanced Amidation for Synergic Detection
of Dopamine and Uric Acid. ACS Omega.

[22] Sivashankari P. R., Prabaharan M. (2020). Three-dimensional porous scaffolds based on agarose/chitosan/graphene
oxide composite for tissue engineering. Int.
J. Biol. Macromol..

[23] Tavakoli M., Karbasi S., Soleymani
Eil Bakhtiari S. (2020). Evaluation of physical,
mechanical, and biodegradation of chitosan/graphene oxide composite
as bone substitutes. Polymer-Plastics Technology
and Materials.

[24] Justin R., Chen B. (2014). Characterisation and drug release
performance of biodegradable chitosan-graphene
oxide nanocomposites. Carbohydr. Polym..

[25] Barra A., Ferreira N. M., Martins M. A., Lazar O., Pantazi A., Jderu A. A., Neumayer S. M., Rodriguez B. J., Enăchescu M., Ferreira P., Nunes C. (2019). Eco-friendly
preparation
of electrically conductive chitosan - reduced graphene oxide flexible
bionanocomposites for food packaging and biological applications. Compos. Sci. Technol..

[26] Yang X., Tu Y., Li L., Shang S., Tao X. M. (2010). Well-dispersed chitosan/graphene
oxide nanocomposites. ACS Appl. Mater. Interfaces.

[27] Pan Y., Wu T., Bao H., Li L. (2011). Green fabrication of chitosan films
reinforced with parallel aligned graphene oxide. Carbohydr. Polym..

[28] Sijie
Wan J. P., Li Yuchen, Hu Han, Jiang Lei, Cheng Qunfeng (2015). Use of Synergistic Interactions to Fabricate Strong,
Tough, and Conductive Artificial Nacre Based on Graphene Oxide and
Chitosan. ACS Nano.

[29] Ping-Ping
Zuo† H.-F. F., Xu Z.-Z., Zhang L.-F., Zhang Yu-L., Xia W., Zhang W.-Q. (2013). Fabrication of biocompatible and mechanically reinforced
graphene oxide-chitosan nanocomposite films. Chem. Central J..

[30] Han
Lyn F., Chin Peng T., Ruzniza M. Z., Nur Hanani Z. A. (2019). Effect
of oxidation degrees of graphene oxide (GO) on the structure and physical
properties of chitosan/GO composite films. Food Packaging and Shelf. Life.

[31] Billing J. F., Nilsson U. J. (2005). Cyclic peptides containing a δ-sugar
amino acid—synthesis
and evaluation as artificial receptors. Tetrahedron.

[32] Sæbø I. P., Bjørås M., Franzyk H., Helgesen E., Booth J. A. (2023). Optimization
of the Hemolysis Assay for the Assessment of Cytotoxicity. Int. J. Mol. Sci..

[33] Stalling S. S., Akintoye S. O., Nicoll S. B. (2009). Development
of photocrosslinked methylcellulose
hydrogels for soft tissue reconstruction. Acta
Biomater.

[34] Tsanaktsidou E., Kammona O., Labude N., Neuss S., Kruger M., Kock L., Kiparissides C. (2020). Biomimetic
Cell-Laden MeHA Hydrogels
for the Regeneration of Cartilage Tissue. Polymers.

[35] Luca
Albertin M. S., Christopher Barner-Kowollik L., Foster John R., Davis T. P. (2004). Well-Defined Glycopolymers from RAFT
Polymerization: Poly­(methyl 6-O-methacryloyl-R-D-glucoside) and Its
Block Copolymer with 2-Hydroxyethyl Methacrylate. Macromolecules.

[36] Kim J. S. H., Park D.-W., Ahn I.-S., Lee T. G., Kim Hae-Sung, Kim Woo-Sik (2004). Biocatalytic esterification
of β-methylglucoside
for synthesis of biocompatible sugar-containing vinyl esters. Chem. Eng. J..

[37] Paslay L. C., Abel B. A., Brown T. D., Koul V., Choudhary V., McCormick C. L., Morgan S. E. (2012). Antimicrobial poly­(methacrylamide)
derivatives prepared via aqueous RAFT polymerization exhibit biocidal
efficiency dependent upon cation structure. Biomacromolecules.

[38] Min
Huang E. K., Lim L. Y. (2004). Uptake and Cytotoxicity of Chitosan
Molecules and Nanoparticles: Effects of Molecular Weight and Degree
of Deacetylation. Pharm. Res..

[39] Guarnieri A., Triunfo M., Scieuzo C., Ianniciello D., Tafi E., Hahn T., Zibek S., Salvia R., De Bonis A., Falabella P. (2022). Antimicrobial
properties of chitosan
from different developmental stages of the bioconverter insect Hermetia
illucens. Sci. Rep.

[40] Basseri H., Bakhtiyari R., Hashemi S. J., Baniardelani M., Shahraki H., Hosainpour L. (2019). Antibacterial/Antifungal Activity
of Extracted Chitosan From American Cockroach (Dictyoptera: Blattidae)
and German Cockroach (Blattodea: Blattellidae). J. Med. Entomol.

[41] Wei Y., Hu X., Jiang Q., Sun Z., Wang P., Qiu Y., Liu W. (2018). Influence of graphene
oxide with different oxidation levels on the
properties of epoxy composites. Compos. Sci.
Technol..

[42] Aranaz I., Alcántara A. R., Civera M. C., Arias C., Elorza B., Heras Caballero A., Acosta N. (2021). Chitosan: An Overview of Its Properties
and Applications. Polymers.

[43] Muda M. S., Kamari A., Bakar S. A., Yusoff S. N. M., Fatimah I., Phillip E., Din S. M. (2020). Chitosan-graphene oxide nanocomposites
as water-solubilising agents for rotenone pesticide. J. Mol. Liq..

[44] Tran M. L., Tran T. T. V., Juang R.-S., Nguyen C. H. (2023). Graphene
oxide crosslinked
chitosan composites for enhanced adsorption of cationic dye from aqueous
solutions. Journal of the Taiwan Institute of
Chemical Engineers.

