# Supplementary material for: Well-Defined Glycopolymer Chitosan Mimics for Design of Chitosan Nanocomposites
Source: Biomacromolecules. 2025 Oct 23;26(11):7755–66. doi: 10.1021/acs.biomac.5c01270 (PMC12606564; doi:10.1021/acs.biomac.5c01270)
Supplement: Supplementary file 1 [file bm5c01270_si_001.pdf]

Supporting Information

Well-Defined Glycopolymer Chitosan Mimics for Design of Chitosan Nanocomposites

*Toby R. Edwards[1], Penelope E. Jankoski[1], Latoyia P. Downs[2], Musa Rabia[2], Lisa K. Kemp[1], Travis L. Thornell[3], Dane N. Wedgeworth[3], J. Kent Newman[3], Tristan D. Clemons[1], Shahid Karim[2], Sarah E. Morgan[1]**

[1]School of Polymer Science and Engineering, University of Southern Mississippi, 118 College Drive # 5050, Hattiesburg, MS 39406

[2]School of Biological, Environmental, and Earth Sciences, University of Southern Mississippi, 118 College Drive #5018, Hattiesburg, MS, 39406

[3]U.S. Army Engineer Research and Development Center, 3909 Halls Ferry Rd, Vicksburg, MS, 39180

^{1}H NMR (D_2O, 600 MHz)

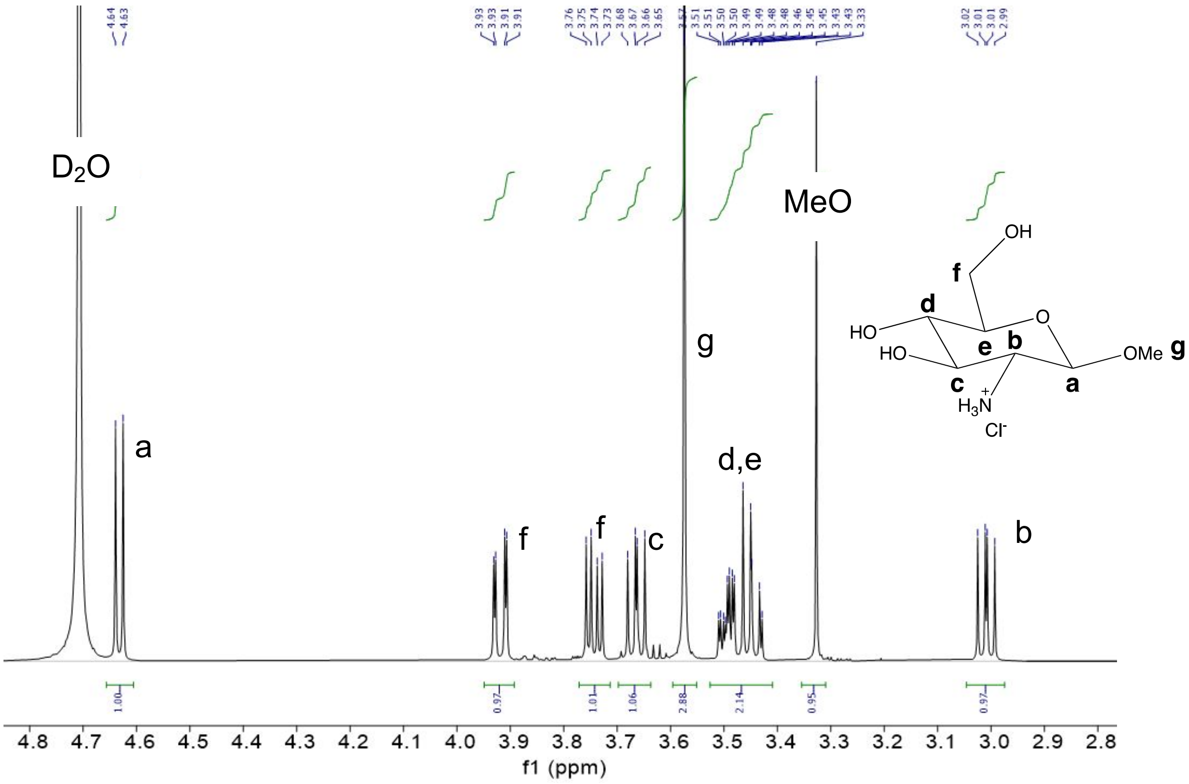

Figure S1. ^{1}H NMR spectrum of methyl glucosaminoside-HCl molecule synthesized using Billing et al. procedure.[1]

^1H NMR (D_2O, 600 MHz)

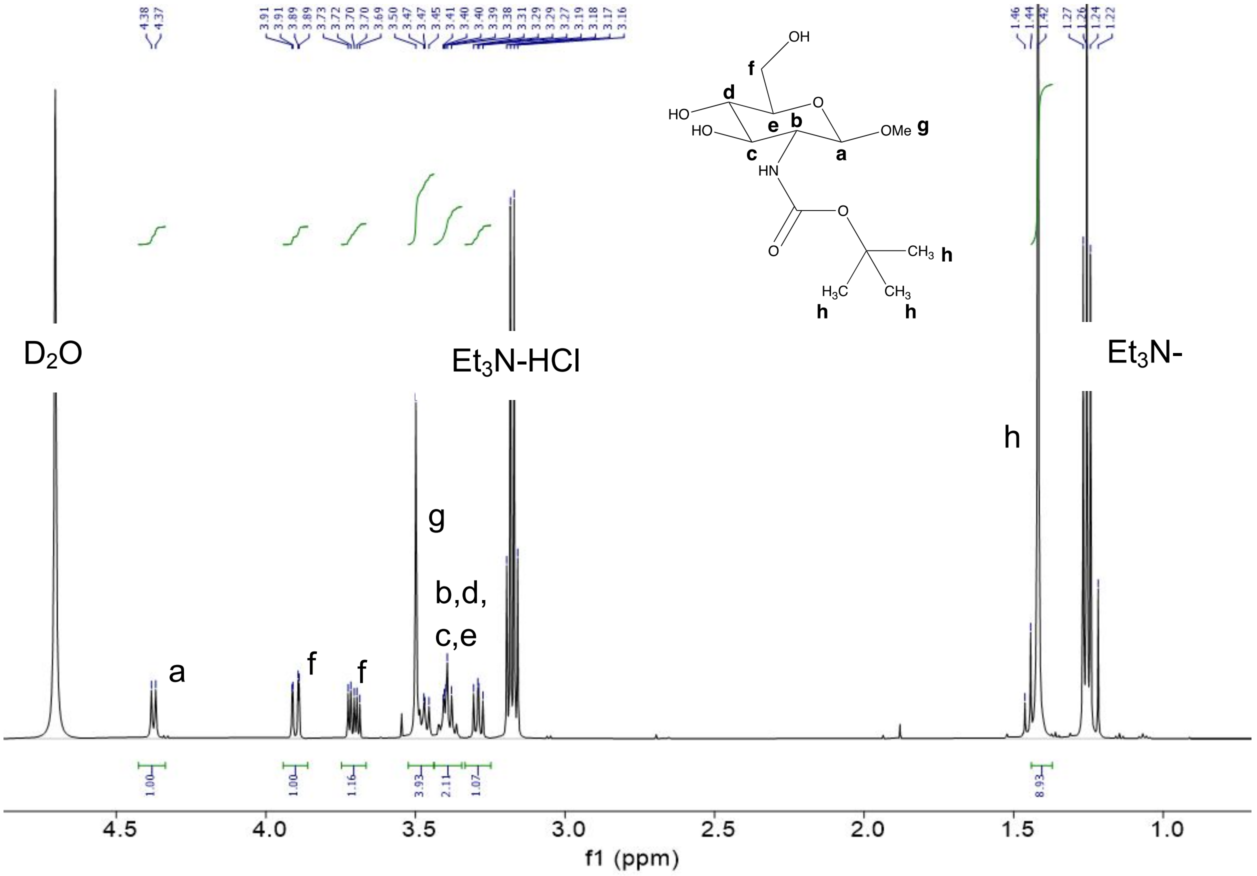

Figure S2. ^1H NMR spectrum of methyl N-boc-β-D-glucosaminoside synthesized using t-boc anhydride and methyl glucosaminoside.

a) ^{13}C NMR (MeOD, 150 MHz)

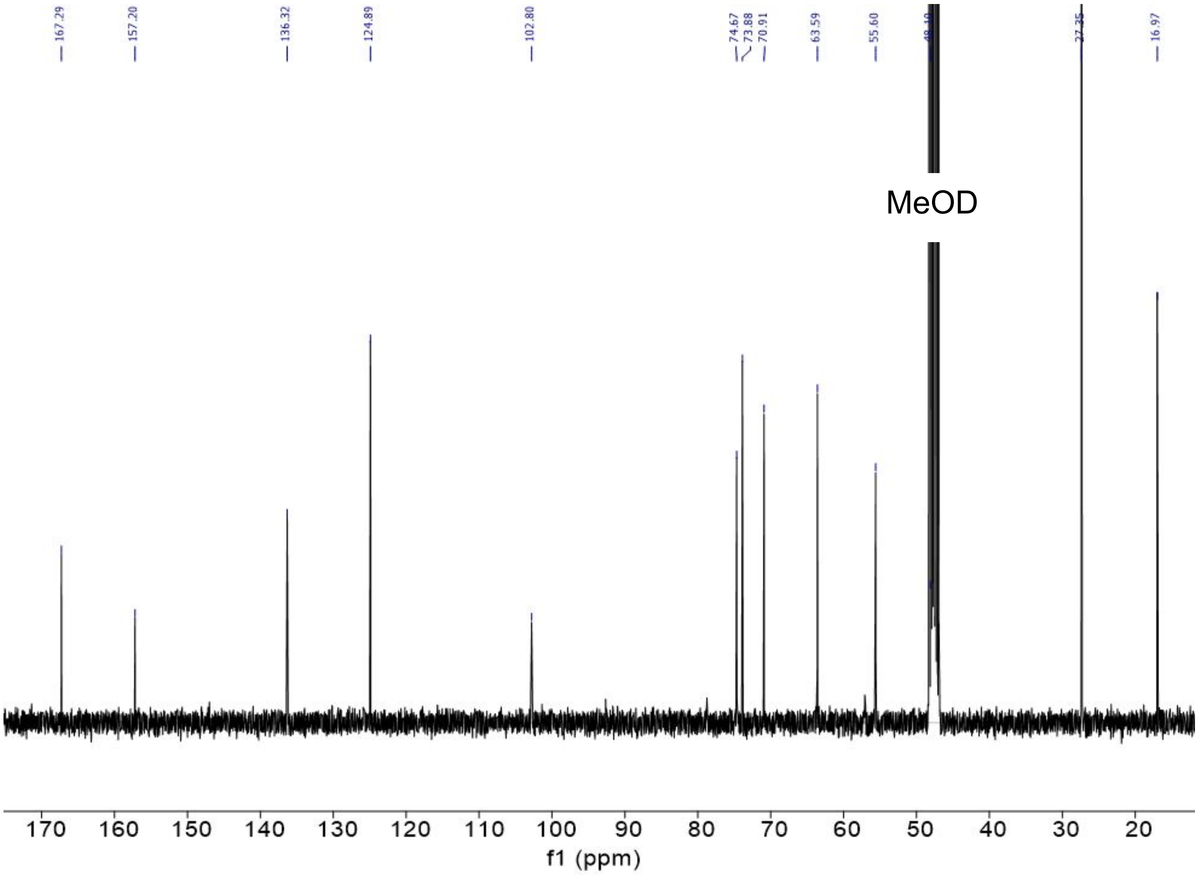

b) ^{1}H NMR (MeOD, 600 MHz)

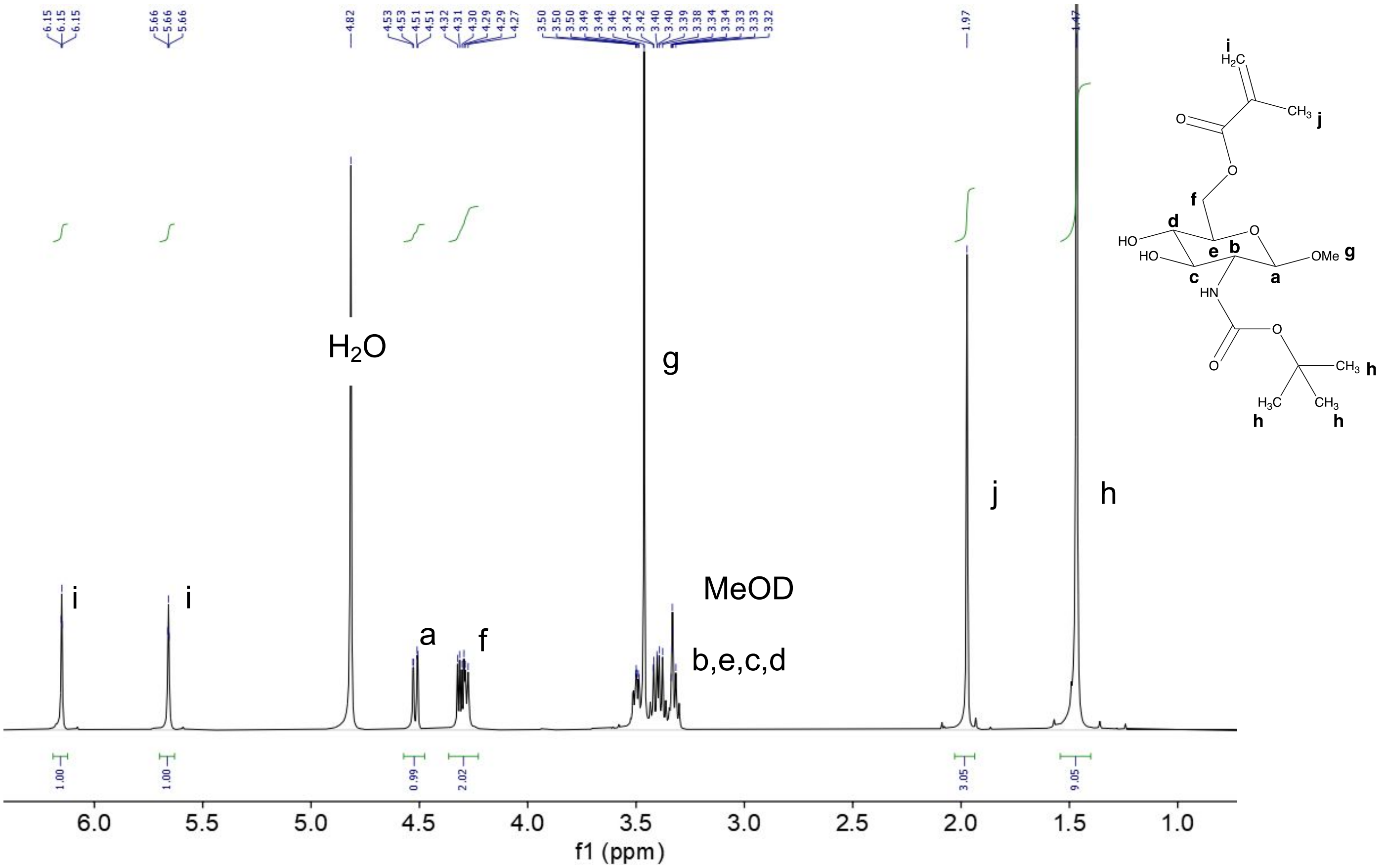

Figure S3. ^{13}C NMR (a) and ^{1}H NMR (b) spectra of methyl N-Boc-6-methacryloyl-β-D-glucosaminoside.

Characterization of Chitosan Mimics.

Deprotected and isolated polymers were characterized using aqueous SEC-MALLS to determine the molecular weight and dispersity (Figure S4).

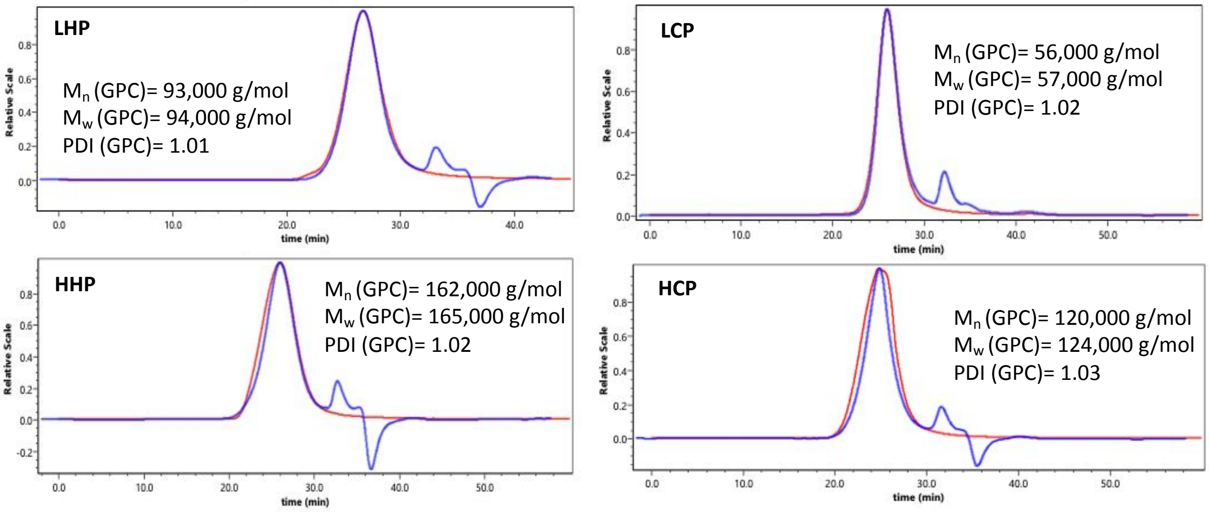

Figure S4. SEC-MALLS traces of LHP, HHP, LCP, and HCP glycopolymer chitosan mimics.

Evaluation of bonding between chitosan mimics and GO.

Four chitosan mimics, synthesized using RAFT polymerization, were blended with samples of GO at two sizes and two reduction levels. A fractional factorial design of experiments (DOE) was used to evaluate the four factors (GO size, GO functionality, mimic M_w, and mimic composition) (2^{4-1}). The design was analyzed in Minitab Statistical Software. Eight combinations of chitosan mimics with GO were evaluated via XPS, and all the samples showed only two peaks in the detailed N1s region of the XPS (Table S1). These values suggest that the combination of chitosan and GO do not form covalent interactions during film formation, and the interactions between the mimics and GO are mainly hydrogen bonding.

Table S1. Fractional factorial DOE design (2^{4-1}) chitosan mimic/GO films were fabricated and studied using deconvolution of the detailed N1s region of XPS.

	GO Size	GO Reduction	Copolymer	Mw	Primary amine (399 eV)	Protonated amine (401.7 eV)
**Sample 1**	nm	No	No	Low	$16 \pm 0.4\%$	84%
**Sample 2**	nm	No	Yes	High	$26 \pm 1.2\%$	74%
**Sample 3**	nm	Yes	No	High	$22 \pm 5.2\%$	78%
**Sample 4**	nm	Yes	Yes	Low	$18 \pm 3.9\%$	82%
**Sample 5**	μm	No	No	High	$23 \pm 1.2\%$	77%
**Sample 6**	μm	No	Yes	Low	$37 \pm 3.6\%$	63%
**Sample 7**	μm	Yes	No	Low	$38 \pm 4.3\%$	62%
**Sample 8**	μm	Yes	Yes	High	$30 \pm 0.4\%$	70%

Table S2. Graphene oxide particle sizes and ratios of carbon to oxygen before and after thermal reduction.

Graphene Oxide Sample	Particle Size Range[a]	Carbon/Oxygen ratio[b]
µm GO	0.5-5µm	1.94
µm rGO	0.5-5µm	6.7
nm GO	90-120nm	1.8
nm rGO	90-120nm	4.2

[a] Size ranges provided by supplier (Graphene Supermarket)

[b] Determined via deconvolution and integration of X-ray photoelectron spectroscopy.

Table S3. Analysis of variance of amine protonation in fractional factorial DOE using Minitab software.

Analysis of Variance

Source	DF	Adj SS	Adj MS	F-Value	P-Value
Model	7	292.373	41.768	0.82	0.599
Linear	4	249.775	62.444	1.22	0.374
Graphene Oxide size	1	18.857	18.857	0.37	0.561
Graphene oxide functionality	1	38.720	38.720	0.76	0.410
MMA percentage	1	98.060	98.060	1.92	0.204
Mimic molecular weight	1	94.139	94.139	1.84	0.212
2-Way Interactions	3	42.598	14.199	0.28	0.840
Graphene Oxide size*Graphene oxide functionality	1	6.878	6.878	0.13	0.723
Graphene Oxide size*MMA percentage	1	4.612	4.612	0.09	0.772
Graphene Oxide size*Mimic molecular weight	1	31.109	31.109	0.61	0.458
Error	8	409.120	51.140		
Total	15	701.493			

Study of Chitosan Film Variability.

Five films were fabricated from the same solution of 3 wt% chitosan in a 1% acetic acid solution to determine the variability in amidation and protonation of chitosan films without the addition of GO. N1s XPS spectra evaluation was performed to determine the percentage of amide and protonated amines in the commercial sample. A range of amide (7 – 12 %, directly related to the degree of deacetylation) and protonated amines (8 – 15%) was observed (Table S3). This natural variation in values could be a source of error leading to wide variation in reported chitosan/GO properties.

Table S4. Amide % and protonated amine % determined via XPS for chitosan films made from a single solution highlighting the variability of films made from the same chitosan sample.

Neat CH Film	Amide % (400.5 eV)	Protonated Amine % (401.7 eV)
Film 1	12%	8%
Film 2	12%	8%
Film 3	10%	14%
Film 4	10%	11%
Film 5	7%	15%

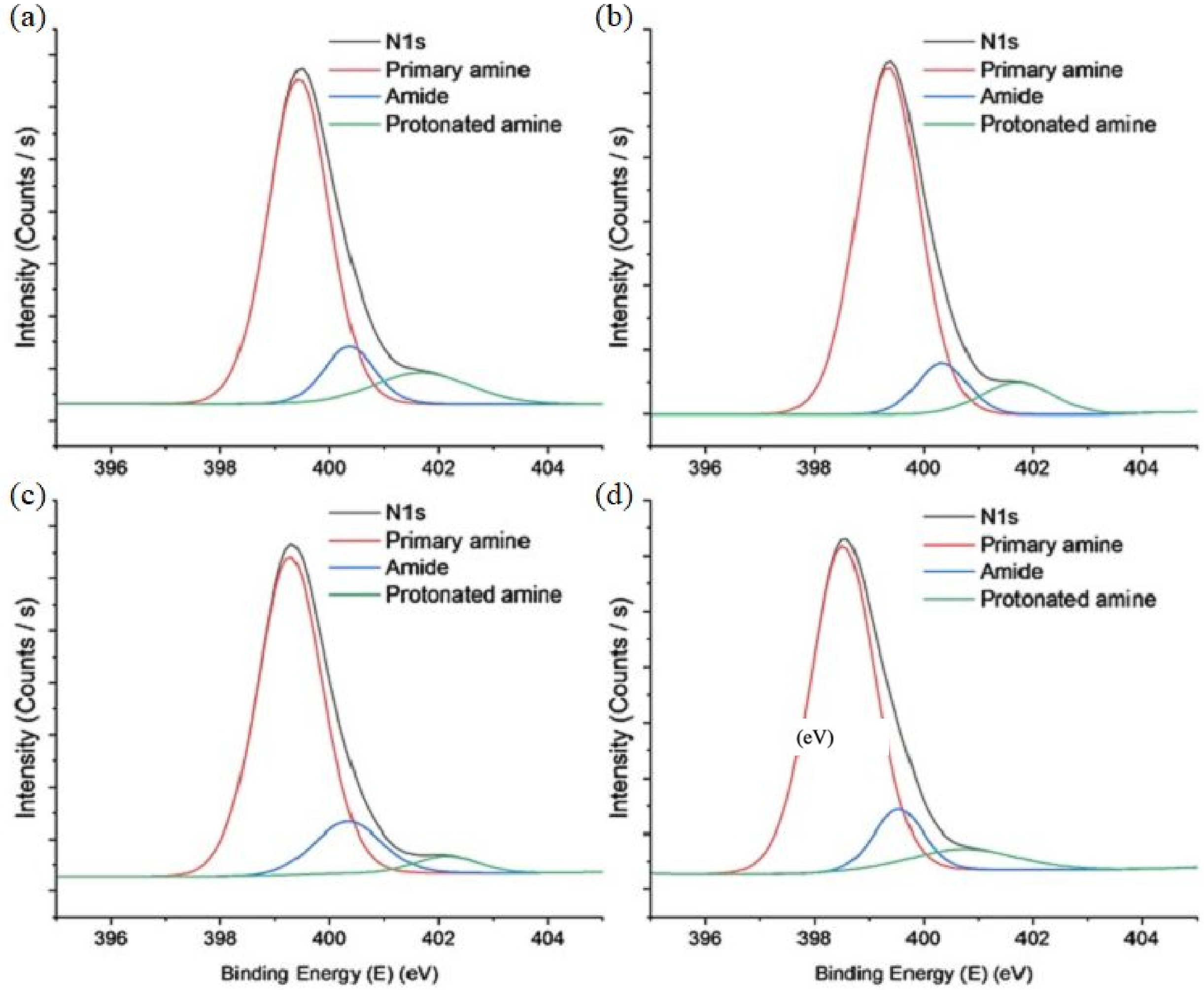

Figure S5. N1s XPS spectra of chitosan/GO films showing three types of nitrogen present for each. (a) CH nm GO (b) CH nm rGO (c) CH μm GO (d) CH μm rGO

1. Billing, J. F.; Nilsson, U. J., Cyclic peptides containing a δ-sugar amino acid—synthesis and evaluation as artificial receptors. *Tetrahedron* **2005,** *61* (4), 863-874.
